# FogFrame: a framework for IoT application execution in the fog

**DOI:** 10.7717/peerj-cs.588

**Published:** 2021-07-05

**Authors:** Olena Skarlat, Stefan Schulte

**Affiliations:** Distributed Systems Group, Technische Universität Wien, Vienna, Austria

**Keywords:** Fog computing, Internet of Things, Service placement, Resource provisioning

## Abstract

Recently, a multitude of conceptual architectures and theoretical foundations for fog computing have been proposed. Despite this, there is still a lack of concrete frameworks to setup real-world fog landscapes. In this work, we design and implement the fog computing framework *FogFrame*—a system able to manage and monitor edge and cloud resources in fog landscapes and to execute Internet of Things (IoT) applications. FogFrame provides communication and interaction as well as application management within a fog landscape, namely, decentralized service placement, deployment and execution. For service placement, we formalize a system model, define an objective function and constraints, and solve the problem implementing a greedy algorithm and a genetic algorithm. The framework is evaluated with regard to Quality of Service parameters of IoT applications and the utilization of fog resources using a real-world operational testbed. The evaluation shows that the service placement is adapted according to the demand and the available resources in the fog landscape. The greedy placement leads to the maximum utilization of edge devices keeping at the edge as many services as possible, while the placement based on the genetic algorithm keeps devices from overloads by balancing between the cloud and edge. When comparing edge and cloud deployment, the service deployment time at the edge takes 14% of the deployment time in the cloud. If fog resources are utilized at maximum capacity, and a new application request arrives with the need of certain sensor equipment, service deployment becomes impossible, and the application needs to be delegated to other fog resources. The genetic algorithm allows to better accommodate new applications and keep the utilization of edge devices at about 50% CPU. During the experiments, the framework successfully reacts to runtime events: (i) services are recovered when devices disappear from the fog landscape; (ii) cloud resources and highly utilized devices are released by migrating services to new devices; (iii) and in case of overloads, services are migrated in order to release resources.

## Introduction

The Internet of Things (IoT) leads to the pervasion of business and private spaces with ubiquitous computing devices, which are able to act autonomously and provide network connectivity (Botta et al., 2016). Together with cloud technologies, the IoT enables small- and large-scale applications for smart cities (Gharaibeh et al., 2017), healthcare (Catarinucci et al., 2015), manufacturing (Compare, Baraldi & Zio, 2020), etc.

IoT data in such applications is mostly produced in a distributed way, sent to a centralized cloud for processing, and then delivered to distributed stakeholders or other distributed IoT devices, often located close to the initial data sources. This centralized processing approach results in high communication latency and low data transfer rates between IoT devices as well as the IoT devices and potential users (Bonomi et al., 2014; Mahmud, Ramamohanarao & Buyya, 2019). Therefore, using centralized resources from the cloud does not match the decentralized nature of the IoT with its bandwidth- and delay-sensitivity. In addition to the centralized processing, the computational resources of IoT devices, which can be used not only for collecting data but also for data processing, are often neglected. Typical examples of such IoT devices which possess computational resources and are capable to host IoT applications are gateways, routers, or sensor nodes (Dastjerdi et al., 2016; Vaquero & Rodero-Merino, 2014; Froiz-Mí­sguez et al., 2018). The combination of edge- and cloud-based computational resources in order to deploy and execute IoT applications is also known as *fog computing*. Together, these resources form a fog computing environment, or a so-called *fog landscape* (Skarlat et al., 2016). Recently, the notions of fog computing were put into the OpenFog Reference Architecture, which became an international standard (IEEE 1934, 2018).

Fog computing has been named as an enabler to provide IoT applications in many different scenarios, especially with regard to smart systems, for example, smart cities, smart buildings, or smart factories (Stojmenovic, 2014; Hu et al., 2017; He et al., 2018; Katona & Panfilov, 2018). By deploying IoT applications in the fog, it is possible, for example, to prefilter data for stream processing or to conduct IoT data processing on-site instead of relying on cloud-based computational resources (Bonomi et al., 2014; Hochreiner et al., 2017). This leads to lower latency in IoT scenarios (Puliafito et al., 2019).

Fog computing has been a vivid field of research in recent years, and the theoretical principles of fog computing as well as conceptual fog architectures are already well-established (Puliafito et al., 2019; Bellendorf & Mann, 2020; Salaht, Desprez & Lebre, 2020; Hong & Varghese, 2019). However, there is a lack of implemented frameworks with the functionality to manage and monitor infrastructure, to deploy and execute services, and to dynamically react to changes in computational demand and fog computing infrastructure.

Notably, fog computing is based on common principles from the field of cloud computing, most importantly virtualization (Yi, Li & Li, 2015; Celesti et al., 2016): while in the cloud, physical machines are provided in terms of virtual machines (VMs), fog computing employs the idea that computational resources from edge devices can be offered in a similar manner. However, since VMs are resource-intensive, they are not the best virtualization approach for rather resource-constraint edge devices (Varghese & Buyya, 2018). A promising solution for this issue is the utilization of *containers*, for example, Docker containers, as a virtualization mechanism for edge resources (Bonomi et al., 2014; Morabito et al., 2018). Accordingly, in order to provide a practical framework for fog computing, it is necessary to introduce mechanisms both to manage fog landscapes and to execute distributed IoT applications in the fog using containers. This requires the provisioning of mechanisms for resource allocation, decentralized service placement, deployment, and execution in a fog landscape.

In this paper, we present the design and implementation of the fog computing framework *FogFrame*. FogFrame is built to provide coordinated control over the physical and virtual infrastructure of a fog landscape. The framework enables volatile IoT landscapes, where the system is ever-changing, with fog nodes and data sources potentially entering or leaving a system at any time, and the data volume to be processed changing frequently (Varshney & Simmhan, 2017; Santos et al., 2019).

Taking into account the potentially volatile nature of fog landscapes, we define the following main goals to be achieved by FogFrame: (i) to create and maintain a fog landscape made up from computational resources at the edge of the network and in the cloud, (ii) to establish communication and interaction in such a fog landscape, (iii) to efficiently deploy and execute IoT applications in the fog by distributing the services of the applications on the available fog resources.

Building on the challenges identified above, we formulate the following research questions that provide the foundation for the work at hand:

- How can edge devices be utilized for the resource-efficient execution of IoT applications?
- How can the execution of IoT applications in a fog landscape be optimized for resource efficiency while considering predefined Quality of Service (QoS) parameters?
- How to achieve a highly available, durable, and fault-tolerant fog landscape?

Our contributions can be summarized as follows:

- We design and implement the FogFrame framework, which provides communication and interaction of virtualized resources within a fog landscape.
- We implement functionalities for decentralized service placement, deployment and execution in a fog landscape. Service placement is performed by two heuristic algorithms—a greedy algorithm and a genetic algorithm. We distinguish service deployment at the edge of the network and in the cloud and implement according deployment mechanisms.
- We develop mechanisms to react to runtime operational events in the fog landscape, namely, when devices appear and disappear in the fog landscape, and when devices experience failures and overloads. The framework identifies those events and migrates necessary services to balance workload between different resources.
- We evaluate the capabilities of FogFrame with regard to service placement, adherence to QoS parameters, and utilization of fog resources.

The work at hand is based on our former work on conceptual fog frameworks and fog computing resource allocation. If compared to our most recent work (Skarlat et al., 2018), this paper reflects in-depth technical details of the architecture of the framework, communication within the fog landscape devices, and the application management. In this work, the framework enables mechanisms to create a fog landscape and account for its volatile nature, namely, it reacts to devices appearing and disappearing from the fog landscape, and tackles overloads and failures of resources. The framework provides a decentralized application execution in the fog and introduces a service placement problem formulation to account for practical issues dealing with volatile fog landscapes. The presented service placement functionalities are based on our former work (Skarlat et al., 2016; Skarlat et al., 2017a, 2017b), where we researched fog computing environments and different service placement approaches and evaluated them using the simulators CloudSim and iFogSim (Gupta et al., 2017). Instead, in this work, we implement and extensively evaluate service placement algorithms in a representative real-world Raspberry Pi-based testbed.

Compared to other frameworks, our framework addresses the volatility of the IoT. We explain and build a real-world fog landscape based on lightweight technologies, and aim at fault-tolerant decentralized application execution and efficient resource provisioning and service placement. As an additional outcome, our framework can be freely used in the research community to develop and evaluate different resource provisioning methods. This is enabled by providing a working software publicly available within our GitHub repository (https://github.com/softls/FogFrame-2.0) for reimplementing exchangeable loosely-coupled components, building and connecting a fog computing environment, and executing IoT applications.

The remainder of this paper is organized as follows: “Fog Landscape Operation” provides the design specifications of FogFrame. Afterwards, “Service Placement” describes the system model for service placement and according placement algorithms. The framework is evaluated in “Evaluation”. “Related Work” discusses the state-of-the-art in the area of fog computing frameworks and service placement algorithms. Finally, conclusions and insights into future work are given in “Conclusion”.

## Fog Landscape Operation

Before describing the needed functionalities of fog computing frameworks, it is necessary to discuss general characteristics of fog landscapes. For this, we follow the notion of the fog as a *thing-to-cloud continuum* (IEEE 1934, 2018).

**Figure 1:**
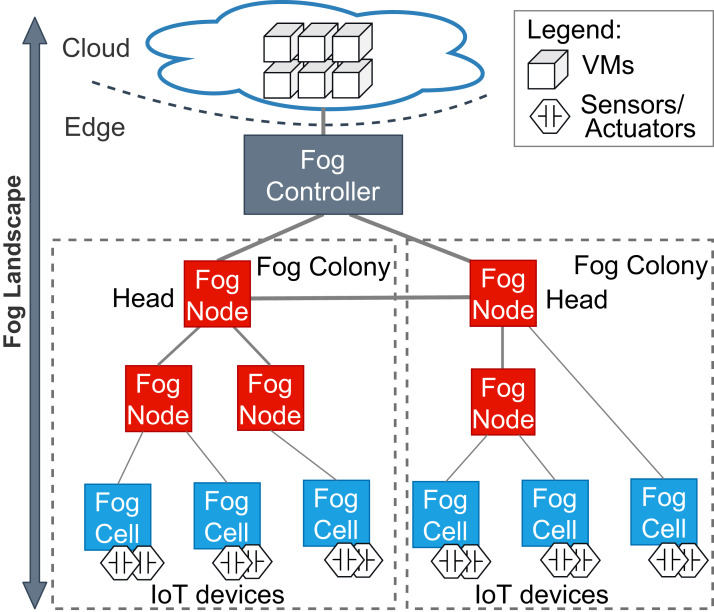
An overview of a fog landscape.

Accordingly, a *fog landscape* consists of the combined computational and storage resource pool of cloud and edge resources (Dastjerdi et al., 2016; Hu et al., 2017). In most state-of-the-art approaches and standardization activities, fog landscapes follow a hierarchical structure (see Fig. 1) (Karagiannis & Schulte, 2021). At the bottom of this hierarchy, there are sensors and actuators, which are attached to different IoT devices. These devices have computational power and are able to host and execute arbitrary services. Within FogFrame, we call such IoT devices *fog cells*. Fog cells control sensors and actuators and are in turn managed and orchestrated by *fog nodes*. Fog nodes are themselves extended fog cells which possess the capabilities to not only host services, but also to perform management activities, such as service placement and deployment. Fog cells and fog nodes are two specific types of *fog devices*.

A hierarchy of fog devices forms a *fog colony*: (i) sensors and actuators attached to fog cells and (ii) fog cells connected to a fog node, which becomes a parent to those fog cells. In each fog colony, there is exactly one head fog node that performs service placement. Other fog nodes can be present in the fog colony, they can perform computations in the same manner as fog cells, and as well be responsible for data communication between the connected fog cells and other fog nodes higher in the hierarchy. Having a hierarchical structure allows to control application deployments over a colony of devices, for example, a fog colony may become a domain-specific execution environment or cover a certain area.

Since fog landscapes comprise computational resources from the cloud and the edge of the network, it is necessary to mediate between fog colonies and the cloud. In FogFrame, we foresee a *fog controller* for this. Head fog nodes communicate with a fog controller in the case additional cloud resources are needed. This controller establishes communication between fog colonies and the cloud. The fog controller also helps to establish communication within and between fog colonies. However, the latter may also act autonomously if the fog controller is not available. Fog colonies are connected to the fog controller via their head fog nodes.

Fog colonies do not only interact with the cloud. Instead, the colonies also need to interact with each other in order to delegate application requests from one colony to another. For instance, if one fog colony does not have enough resources to execute an application, then it may delegate the corresponding application request to a neighbor fog colony. To do this, fog colonies are connected to each other via their corresponding head fog nodes.

To establish coordinated control over a fog landscape, a fog computing framework has to be able to monitor and control the available devices and computational resources in the cloud and at the edge and to orchestrate those resources in order to deploy arbitrary services. Hence, FogFrame needs to provide the following functionalities: (i) Mechanisms to manage and support a fog landscape, namely, to establish communication within the fog landscape and to handle data transfers between fog colonies and the cloud; (ii) Mechanisms to manage application execution in an efficient manner by optimizing resource provisioning and service placement; and (iii) Methods to automatically migrate services due to the volatile nature of the fog landscape, for example, because new resources at the edge of the network are discovered, already existing resources become overloaded, or even disappear from the fog landscape due to failures. In the following subsections, we will describe how FogFrame provides these functionalities.

### Communication

A fog landscape starts with instantiating a fog controller, which is an initial communication point for fog colonies. After instantiating the fog controller, fog devices can enter the fog landscape and start forming fog colonies. When creating the fog landscape, we follow the assumptions that (i) all fog devices are able to provide their location data, (ii) all fog devices are configured with the fog controller address to request joining the fog landscape (or are able to get the fog controller IP addresses through some bootstrapping mechanism), and (iii) all fog nodes can operate within predefined coverage areas, can form fog colonies, and operate fog devices within their coverage area.

To enter the fog landscape, a fog device sends an asynchronous pairing request containing the own location data to the fog controller. The fog controller has a dedicated location service that based on the location coordinates and coverage areas of all fog nodes in the fog landscape returns data about the fog node that becomes a parent to this fog device. This is possible because each fog device contains data about its own device name, IP address, and location coordinates. Other data differs according to the fog device type, for example, fog nodes also contain a coverage area parameter defined. The coverage area defines a geographical area each fog node is responsible for. The criteria of finding a parent fog node could be based on different aspects, for example, the calculation of the physical distance between fog devices, but also efficiency, ratio of successful service execution, or latency. For the purposes of FogFrame, we have implemented searching for the closest parent according to the location of the fog device which enters the fog landscape, but it would be possible to extend this functionality by the criteria just mentioned.

**Figure 2:**
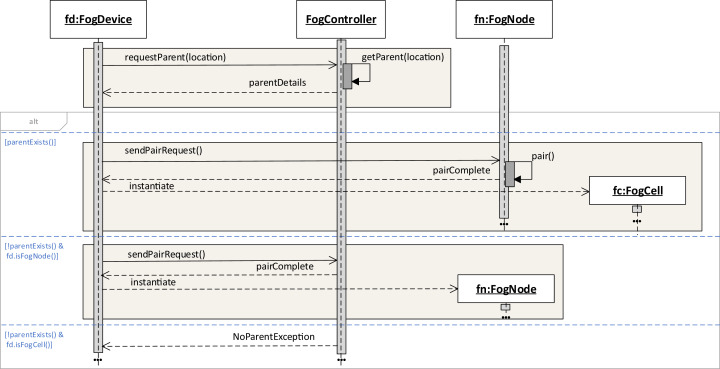
Instantiating fog cells and fog nodes in a fog landscape.

If the request is satisfied, the fog device sends a pairing request to this fog node. Upon successful pairing, the device is instantiated as a fog cell or as a fog node in the fog colony and is added to the set of children of the fog node. If the request is not satisfied, we consider two possible outcomes: (i) if the fog device is a fog cell, an error message is returned, and (ii) if the fog device is a fog node, this fog node becomes the head of a new fog colony as it has a unique range of location coordinates, namely, its coverage area. This workflow is shown in Fig. 2.

To be able to delegate applications to a neighbor fog colony, each head fog node has to be connected with the head fog node of a neighbor fog colony. This connection is also established when a fog node is instantiated in the fog landscape. It requests the neighbor head fog node from the fog controller. The location service of the fog controller finds the closest neighbor head fog node according to the provided location coordinates. If the request is satisfied, the head fog node sends a pairing request to the closest neighbor head fog node. If the request is not satisfied, the head fog node either connects with a fallback neighbor fog colony, or continues to act autonomously.

Upon joining a fog colony, it is the goal that a request from a fog cell to the direct parent, which is a fog node, can be satisfied. If a pairing request cannot be satisfied, a fallback mechanism is applied. For that, we enable searching for a fallback parent or grandparent fog node if the closest fog node or even the fog controller are not available. Fallback details can be either implemented as an internal property of a device, or can be sent to the device upon pairing. In FogFrame, fallback parent and grandparent IP addresses are provided as properties of each fog device.

### Application management

**Figure 3:**
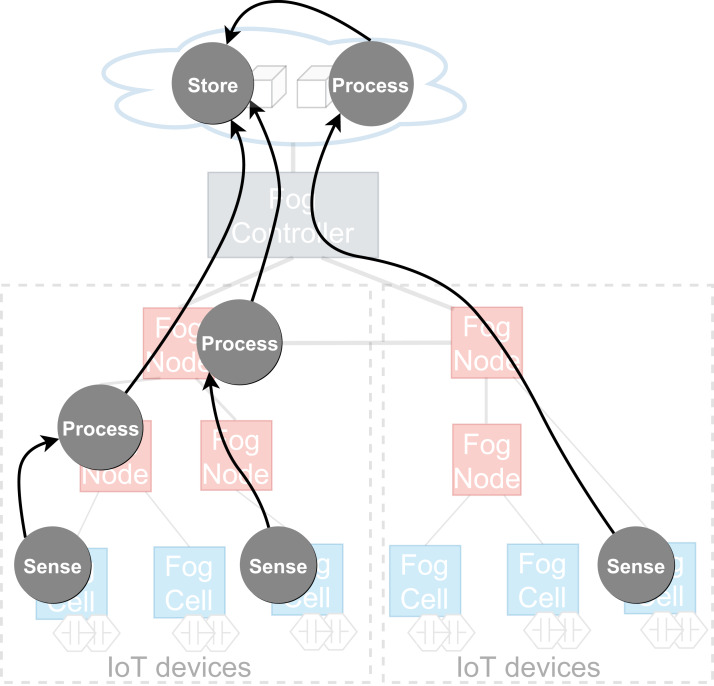
An example of a distributed data flow in a fog landscape.

To achieve cooperative execution of IoT applications, a fog landscape has to enable decentralization of application execution, making it possible that different parts of an IoT application are deployed and executed close to the relevant data sources and data sinks. Because of the benefits of containerized applications mentioned in “Introduction”, in FogFrame, *applications* are built following the microservice architectural approach from stateless services deployed and executed to achieve a certain result. An application can be visualized as a distributed data flow (Giang et al., 2015)—a directed acyclic graph where vertices are tasks to be executed in the flow, corresponding to services, and edges between vertices are data shipment connections between those services (Kougka & Gounaris, 2019) (see Fig. 3). *Services* are deployed and running computational software instances which process service requests in the fog landscape. A *service request* is a single computational job to be computed on fog devices. Services can be of certain *service types*. Service types are bound to the capabilities of the devices in the fog landscape. For example, a service intended to receive temperature measurements can be deployed only on a fog cell with a temperature sensor attached, some services can be executed either in the cloud or in the fog, and other services can be executed only in the cloud.

**Figure 4:**
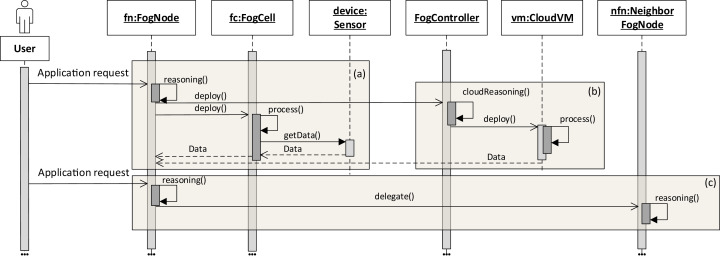
Application request processing on different fog resources (A–C).

The application execution starts with an *application request* which defines a set of services to be placed and deployed in the fog landscape together with QoS information for execution, for example, deadlines on application execution and processing times. It has to be noted that an application can only be executed if all its services are deployed. The deployment of services depends on the service placement mechanism which is applied within each head fog node of each fog colony in the fog landscape. It is possible to integrate arbitrary service placement algorithms into FogFrame. Within the work at hand, we provide two particular approaches aiming at utilizing available resources of fog colonies in the most efficient way, as presented in “Service Placement”. In the following, we describe how the application is processed on different resources in the fog landscape: inside a fog colony, in the cloud, or after being delegated to a neighbor fog colony (see Fig. 4).

Application deployment and execution can be done based on different settings. The first (and simplest) setting is when each fog colony has enough own resources to execute an application request. In this case, all the necessary services are deployed in the current fog colony. The latency and deployment time are minimal and depend on the computational power of the resources of the colony. As it can be seen in Fig. 4A, a user submits an asynchronous application request to a fog node. The service placement is performed according to the chosen service placement algorithm by the reasoning service of the fog node. It has to be noted that an application request can also be submitted to a fog cell (not depicted in Fig. 4). If this is the case, the fog cell forwards the request to its parent fog node until the request reaches the head fog node of the corresponding fog colony, which performs service placement.

Service placement is performed in a decentralized manner and is independent in each fog colony. To perform computations, the reasoning service uses information about the availability and utilization of all the fog cells in the fog colony. The result of the calculations in the algorithms is a *service placement plan*. After the service placement plan is calculated, the head fog node deploys the necessary services on according fog cells, and the fog cells immediately start service execution.

The second setting of application execution is when apart from executing services in a fog colony, it is necessary to support fog colonies with additional resources from the cloud (Figs. 4A–4B). This applies for services which can be executed only in the cloud, for example, big data processing, and those services which cannot be placed on fog devices because of QoS constraints or a lack of resource capabilities, or services which can be executed either on the edge devices or in the cloud as they do not require specific sensor equipment. If specific services in the application request are assigned to the cloud, the fog node sends the request to execute this service in the cloud. For this, the fog controller authenticates itself with the cloud provider, either leases and instantiates a new VM in the cloud or connects to an existing VM, and deploys the corresponding service container. Specific implementation details about service deployment at the edge of the network and in the cloud are provided in “Evaluation”.

The third setting of application execution regards if an application cannot be executed by a fog colony. However, this application requires sensor equipment, therefore it cannot be executed purely in the cloud. In this case, the request is delegated to a neighbor fog colony. This is the case when the service placement plan determines that there are not enough resource capacities in the current fog colony to execute the application, however there is enough time indicated by the application deadline to postpone the execution. Therefore, the fog node delegates the application request to the neighbor fog colony (Fig. 4C).

### Migration of services

As has already been mentioned above, running an application is only possible when all its services are deployed. Hence, if a fog device fails, services which have been running on that fog device need to be redeployed on other fog devices to ensure the application execution. If a new fog cell appears, it is beneficial to use its capacities to release other devices that are not yet overloaded but already close to full capacity, and migrate suitable services from the cloud to reduce additional unnecessary cost of fog landscape operation. To address this, FogFrame can react to certain events: (i) device discovery, if a fog device appears in the fog landscape, (ii) device failure, if a fog device is not able to provide services any longer, and (iii) device overload, if a fog device is expected to overload, meaning when the CPU, RAM or storage utilization is above a predefined threshold. To handle each of these event types, FogFrame implements corresponding services. To achieve this, each fog device is provided with a *host monitor service* which records CPU, RAM and storage utilization of each device. Correspondingly, each fog node contains a *watchdog* which periodically checks if the connected fog devices respond.

A common event in a fog landscape is device overload. When the CPU power is used up to the maximum specified level, unexpected performance can take place and compromise the execution of all deployed services. In this work, we aim for 80% CPU load to get a balance between utilization and room for spikes, ad-hoc processes, and I/O bottlenecks. This threshold was set up during pre-experiments to allow for uninterrupted execution and availability of the devices. This threshold is also recommended by AWS (https://docs.aws.amazon.com/autoscaling/ec2/userguide/as-scaling-simple-step.html and Oracle (https://docs.oracle.com/en/cloud/get-started/subscriptions-cloud/mmocs/setting-alert-performance-metric.html). This parameter can be easily changed in the FogFrame monitoring component. It is a matter of future research to take into account different thresholds for different devices according to their capabilities, namely, adaptive thresholds as presented, for example, by Maurer, Brandic & Sakellariou (2013).

When a device is identified as overloaded by the head fog node, this event is therefore triggered and the device *overload service* triggers service placement (as presented in “Application Management”) bounded to the current fog colony, and migrates one random container from the affected device. The device overload service migrates services one by one until the affected device is not overloaded anymore. This migration method was adopted according to similar techniques in cloud computing (Smimite & Afdel, 2020). The overload policy can be easily reimplemented and substituted with other methods. The framework architecture is loosely coupled and allows to add new implementations of used methods. A very promising approach to predict overload of fog devices presented by Nair & Somasundaram (2019) could be implemented in future work.

If a new fog device joins a fog colony, all its resources are analyzed to identify the current workload. First, if applicable, the discovery service migrates the services from the cloud to save cost. Second, all devices that operate at the maximum capacity and are overloaded, are considered for migration. The *discovery service* triggers the placement of suitable services from overloaded devices and migrates them to the new device either while needed, or until it is filled to a maximum defined number of containers. The number of containers can be adjusted according to the computational capacity of each fog device. This mechanism allows easy horizontal scalability of the resources in the fog landscape.

It has to be noted that the vertical scalability of resources is bounded by the computational capacities of devices in a fog landscape: If it is possible to extend CPU or RAM on a device, then FogFrame will accordingly use those capacities. This is enabled by the implemented host monitor service that is deployed as a satellite service to each fog cell or fog node. This monitoring service will be described in the next subsection.

Due to the volatile nature of fog landscapes, fog devices may disappear from the fog landscape when a physical IoT device gets out of range or disconnects. When a fog cell disappears from a fog colony due to a failure, the head fog node checks the service assignments to identify whether the failed fog device had any services running. The *device failure service* adds all services that were running on that fog device to a migration list, and triggers service placement as presented in “Application Management”, bounded to the current fog colony. Failures of fog cells are identified by a parent fog node when the fog cell is disconnected. Failures of fog cells trigger the migration service. Because fog nodes take care of communication between their connected fog cells and other fog nodes higher in the fog colony’s hierarchy, a failure of such an intermediary fog node not only triggers the migration service to recover all running services, but also requires fog cells to ask for a new parent fog node from the fog controller. In the case when the head fog node of the fog colony fails, then either there is a mechanism in place to connect all the fog colonies resources to a fallback head fog node, or to trigger a complete reorganization of the fog colonies. The latter is a topic for future research, that can be formulated as a meta control mechanism for reactive and proactive reorganization and optimization of fog colonies to ensure system durability and fault tolerance.

### System architecture

To summarize the discussion of the system design, we discuss the high-level architecture of the main components of FogFrame, namely the fog controller, fog nodes, and fog cells. These components consist of dedicated services and interfaces which provide communication between the components, and within the components as well. The design of the framework is based on lightweight technologies and loosely-coupled components with the goal to create a stable and fault-tolerant distributed system. The extensible modules within each component enable interoperability and a convenient substitution by implementing the specified interface methods.

**Figure 5:**
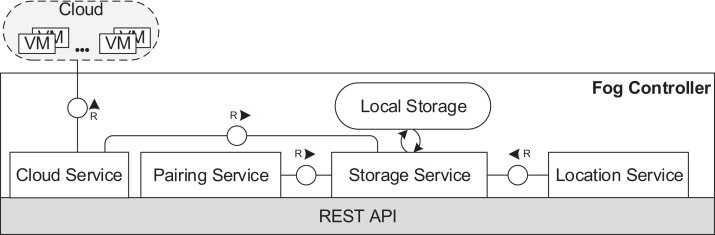
Fog controller architecture.

In the fog controller (see Fig. 5), the *cloud service* establishes the communication with the cloud and implements necessary functionalities to manage VMs and containers. The *location service* provides connection data for fog devices entering the fog landscape. The *pairing service* is responsible for pairing of fog devices as described in “Communication”. A *local storage* stores data about the structure of the fog landscape and the usage of cloud resources. The local storage is operated by the *storage service*.

**Figure 6:**
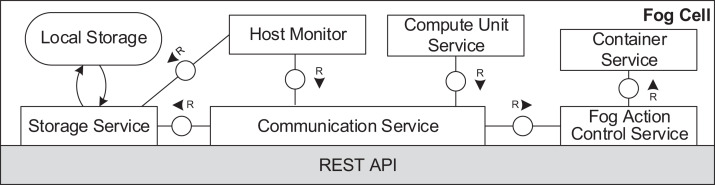
Fog cell architecture.

**Figure 7:**
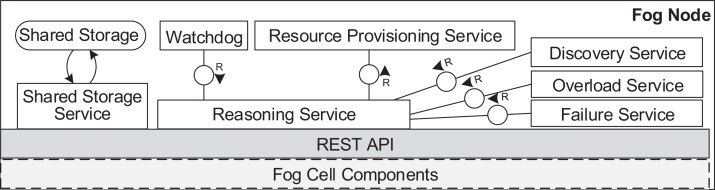
Fog node architecture.

Figures 6 and 7 depict the architectures of fog cells and fog nodes. All components are communicating via API calls. As it can be seen, fog nodes are extended fog cells. The fog cell consists of a *storage service* which operates a *local storage*, storing connection data, identification data, and application execution data. The *communication service* is responsible for establishing and maintaining communication with the fog controller and the parent fog node. The *compute unit* executes services and is responsible for data transfer between services. The *fog action control* follows the orders from the fog node of the fog colony, and deploys necessary services by the means of the *container service*. Finally, the *host monitor* monitors the utilization of the fog cell.

A fog node consists of all the components of a fog cell as well as some additional components. The *shared storage service* operates the *shared storage* which stores a *shared service registry* of all service images to be used for deployment. The local storage on the fog node is similar to the one in the fog cell. However, it stores additional data about fog cells in the fog colony, service placement plans, and execution details of applications. The shared storage service in a fog node is intentionally separated from the local storage container to ensure flexibility and replaceability. The *watchdog* constantly monitors the utilization data of all the connected fog devices, and triggers runtime events: device discovery, overload or failure events. The *reasoning service* is triggered when an application request is submitted for execution to the fog node. The reasoning service calls the *resource provisioning service*, which implements a certain service placement algorithm. We will present two particular algorithms for this in the next section, but as pointed out above, any arbitrary placement algorithm could be applied here. To summarize, the fog cell and the fog node provide all necessary functionalities to establish application management and account for the volatile nature of the fog landscape. Implementation details are provided in “Evaluation”.

## Service Placement

FogFrame allows decentralized service placement, which is ensured by reasoning and placement capabilities of each head fog node in fog colonies, as has already been mentioned in “Fog Landscape Operation”. Fog colonies autonomously perform service placement, even in case the fog controller or cloud-based computational resources are not available. The underlying service placement model, which will be discussed in detail in this section, determines an optimal mapping between services of applications and computational resources at the edge of the network and in the cloud (if available). The resource provisioning and service placement problem has been shown to be NP-hard (Afzal & Kavitha, 2019). For this problem, an analogy towards the multiple knapsack problem can be performed (Amarante et al., 2013): different fog resources are knapsacks, single services of IoT applications are items to be inserted into knapsacks, the weight of knapsacks corresponds to available resources of fog devices, such as CPU, RAM and storage, and the costs of the knapsack are the defined QoS parameters. The complexity of a multiple knapsack problem is proven to be *O*(*n*^2^ + *nm*) (Detti, 2009), where *n* is the number of services to be placed and *m* is the number of fog resources available in a fog colony. For service placement, the objective function is to maximize the utilization of devices at the edge of the network while satisfying the QoS requirements of applications, namely, satisfying deadlines on application deployment and execution time.

For this, every head fog node considers resources available in its fog colony, cloud resources, and the closest neighbor fog colony. A mapping between applications and computational resources determines the following subsets of service placement: (i) services to be executed on fog devices in the fog colony, (ii) services to be executed locally on the fog node, and (iii) services to be executed in the cloud. If at least one service of the application cannot be placed in those subsets, the whole application request is sent to the closest neighbor fog colony. Splitting single services from applications and delegating them to the neighbor fog colony is not considered in order to eliminate tracking of single services in the fog landscape and high intra-application latency and because of the necessary coordination between fog colonies. The service placement approach is reactive, namely, whenever application requests are submitted for execution to a fog node, the service placement algorithm as described in “Application Management” is triggered. Additionally, service placement is triggered each time operational runtime events happen in the fog landscape: appearing and disappearing of resources at the edge, failures and overloads. In the following, we formalize the according system model. Table 1 gives an overview of the notation of fog resources and applications.

**Table 1:** Notation of the fog resources and applications.

Notation	Definition
	**Fog resources**
*t*	Current placement time
*τ*	Time difference of previous placement
*R*	Cloud
*F*	Head fog node
*N*	Closest neighbor to *F*
*Res*(*F*)	Fog cells connected to F
*C* = {*U*, *M*, *S*}	Resource capacities of fog devices
*U*_*F*_	CPU capacity of *F*
*M*_*F*_	RAM capacity of *F*
*S*_*F*_	Storage capacity of *F*
*K*_*F*_	Container capacity of *F*
*f*_*j*_	Fog cell
*U*_*f_j_*_	CPU capacity of *f*_*j*_
*M*_*f_j_*_	RAM capacity of *f*_*j*_
*Sf_j_*	Storage capacity of *f*_*j*_
*K*_*f_j_*_	Container capacity of *f*_*j*_
*d*_*f_j_*_	Latency between *F* and *f*_*j*_
*d*_*R*_	Latency between *F* and *R*
*d*_*N*_	Latency between *F* and *N*
	**Application**
*A*	Set of applications to be executed
*A*_*k*_	Application
*D*_*A_k_*_	Deadline of *A*_*k*_
*w*_*A_k_*_	Deployment time of *A*_*k*_
*w*^*t*^_*A_k_*_	Already passed deployment time of *A*_*k*_ at *t*
\overline T _{{w_N}}^t	Deployment time in the neighbor colony
*m_A_k__*	Makespan duration of *A*_*k*_
*r*_*A*_*k*__	Response time of *A*_*k*_
|*A*_*k*_|	Number of services in *A*_*k*_
*a*_*i*_	Service in an application *A*_*k*_
*U*_*a*_*i*__	CPU demand of service *a*_*i*_
*M_a_i__*	RAM demand of service *a*_*i*_
*S_a_i__*	Storage demand of service *a*_*i*_
*m_a_i__*	Makespan duration of service *a*_*i*_
*Res_a_i__* (*F*)	Fog cells able to host service *a*_*i*_

### System model

#### Domain definition

The decision variables *x*^*f_j_*^_*a_i_*_, *x*^*F*^_*a*_*i*__, *x*^*R*^_*a*_*i*__ indicate the placement of a service *a*_*i*_ on a specific resource in a fog landscape, namely, on a fog cell *f*_*j*_, fog node *F*, or in the cloud *R*. The decision variable *y_A_k__* indicates that the request for the execution of the application *A*_*k*_ has to be delegated to the closest neighbor fog colony, with the head fog node *N*. Together, the decision variables form a *service placement plan*.

Let *a*_*i*_ denote a service of the application *A*_*k*_. In order to ensure that a service is compatible with the allocated resource, *Res_a_i__*(*F*) is introduced to denote all the fog cells capable to run service *a*_*i*_, with }{}Re{s_{{a_i}}}(F) \subseteq Res(F). This formalism is necessary to account for service types, with which resources can be compatible with, for example, sensing, processing service types. The decision variables of the service placement problem are provided in Eqs. (1)–(4):

(1) }{}x_{{a_i}}^{{f_j}} \in \{ 0,1\} ,\forall {a_i} \in {A_k},\forall {f_j} \in Re{s_{{a_i}}}(F)

(2) }{}x_{{a_i}}^F \in \{ 0,1\} ,\forall {a_i} \in {A_k}

(3) }{}x_{{a_i}}^R \in \{ 0,1\} ,\forall {a_i} \in {A_k}

(4) }{}{y_{{A_k}}} \in \{ 0,1\}

#### Objective function

The objective function of the service placement is to maximize the number of service placements in the available fog colonies, while satisfying the QoS requirements of applications, as defined in (5). Unlike execution of all services within one fog colony, delegation of the application to the closest neighbor fog colony or execution in the cloud suggests additional delays, which can become a serious constraint when the response time of the application is close its declared deadline. Hence, we use the prioritization coefficient *P*(*A*_*k*_) for each application. The coefficient *P*(*A*_*k*_) represents a weight of an application *A*_*k*_ determined by the the difference between the deadline *D*_*A_k_*_ of the application and its already recorded deployment time *w_A_k__*, as defined in (6). *w_A_k__* appears in the cases when the application was propagated from another fog colony and had to wait until it is correctly placed on necessary resources. The priority for deployment is given to the applications with high *w_A_k__*, and accordingly little difference *D_A_k__* − *w_A_k__*. *N*(*A*_*k*_) denotes the number of services in the application request to be placed in fog colonies, as defined in (7).

(5) \max \sum\limits_{{A_k}}^A P({A_k})N({A_k})

(6) }{}P({A_k}) = \displaystyle{1 \over {{D_{{A_k}}} - {w_{{A_k}}}}}

(7) }{}N({A_k}) = \sum\limits_{{a_i}}^{{A_k}} \left( {\sum\limits_{{f_j}}^{Re{s_{{a_i}}}(F)} x_{{a_i}}^{{f_j}} + x_{{a_i}}^F} \right) + |{A_k}|{y_{{A_k}}}

#### Constraints

The first set of constraints defines the usage of available CPU, RAM, and storage of fog resources (*U_a_i__*, *M_a_i__*, and *S_a_i__*, respectively). The sum load of placed services should be within the tolerance limits of resource capacities of according fog devices, as shown in (8)–(10). The tolerance limit of a resource is *γ* ∈ [0,1], for example, *γ* = 0.8 indicates that 80% of all device resources can be used to execute services and the rest 20% should be kept free in order to account for operational stability of the device. As described in “Migration of Services”, it would also be possible to utilize an approach based on adaptive thresholds. *C* = {*U*,*M*,*S*} denotes the corresponding capacities of CPU, RAM, and storage of fog cells and the fog node, and the corresponding CPU, RAM, and storage demands of services (see Table 1).

(8) \sum\limits_{{A_k}}^A \sum\limits_{{a_i}}^{{A_k}} {C_{{a_i}}}x_{{a_i}}^{{f_j}} \le \gamma {C_{{f_j}}},\forall {f_j} \in Re{s_{{a_i}}}(F)

(9) \sum\limits_{{A_k}}^A \sum\limits_{{a_i}}^{{A_k}} {C_{{a_i}}}x_{{a_i}}^F \le \gamma {C_F}

(10) }{}C = \{ U,M,S\}

To execute applications according to the necessary QoS, the response time *r_A_k__* of each application *A*_*k*_ has to be less that the declared deadline *D*_*Ak*_ of the application, as defined in (11). The response time *r_A_k__* is defined as the sum of the total makespan duration *m_A_k__* and its deployment time *w_A_k__*, as defined in (12).

(11) }{}{r_{{A_k}}} \le {D_{{A_k}}},\forall {A_k} \in A

(12) }{}{r_{{A_k}}} = {m_{{A_k}}} + {w_{{A_k}}}

The total makespan duration *m_A_k__* consists of execution times of all single services of the application *A*_*k*_ accounting for the according communication delays between the head fog node and chosen for placement resources, as defined in (13)–(14).

(13) }{}{m_{{A_k}}} = \sum\limits_{{a_i}}^{{A_k}} {L_{{a_i}}}

(14) \matrix{ {{L_{{a_i}}} = } \hfill & {\sum\limits_{{f_j}}^{Re{s_{{a_i}}}(F)} d({a_i},{f_j})x_{{a_i}}^{{f_j}} + d({a_i},F)x_{{a_i}}^F + d({a_i},R)x_{{a_i}}^R} \hfill \cr }

*d*(*a*_*i*_,*f*_*j*_), *d*(*a*_*i*_,*F*), and *d*(*a*_*i*_,*R*) denote the makespan duration *m_a_i__* of a service *a*_*i*_ in the case of its placement and execution on the fog cell *f*_*j*_, fog node *F*, or cloud *R* respectively ((15)–(17)).

(15) }{}d({a_i},{f_j}) = {d_{{f_j}}} + {m_{{a_i}}}

(16) }{}d({a_i},F) = {m_{{a_i}}}

(17) }{}d({a_i},R) = 2{d_R} + {m_{{a_i}}}

**Figure 8:**
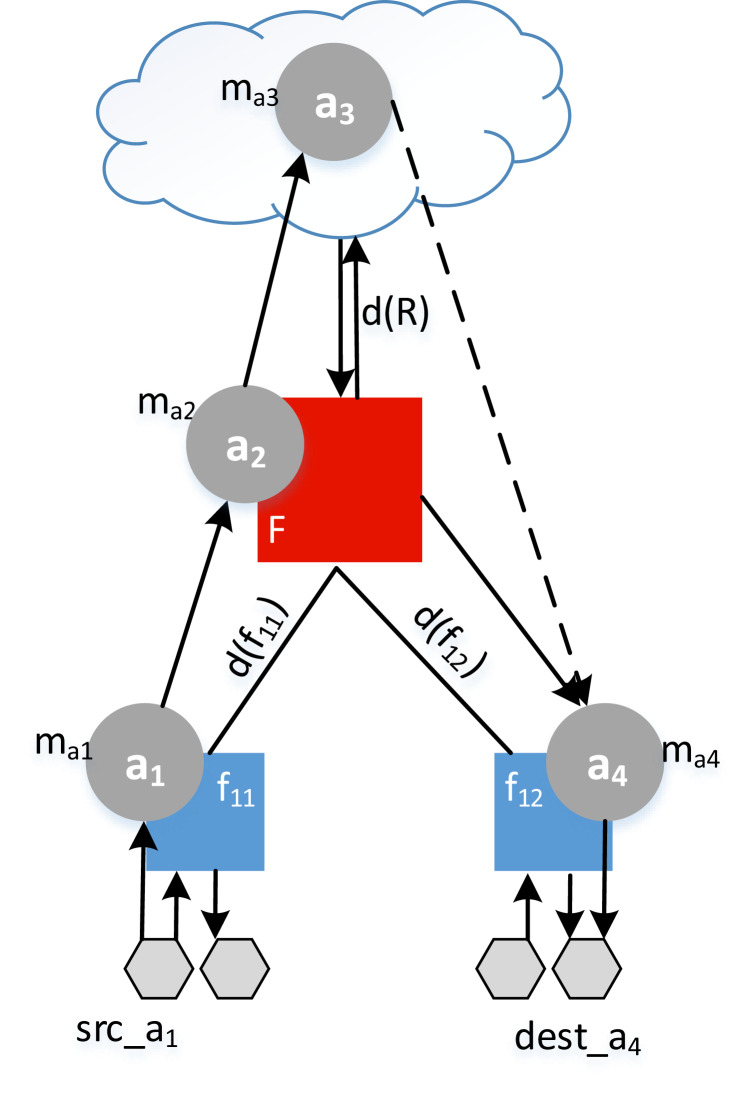
Example of a response time calculation.

*Example*. Let the application *A*_1_ = {*a*_1_,*a*_2_,*a*_3_,*a*_4_} be distributed between a fog colony and the cloud (see Fig. 8). To find the response time of an application *r*_1_, the makespan of each service *m_a_i__* is added to the delay and summed as in (18). In this example, we assume that the application had no previous deployment time, so that *w_A_1__* = 0.

(18) }{}{r_1} = d({f_{11}}) + {m_{a1}} + {m_{a2}} + 2d(R) + {m_{a3}} + d({f_{12}}) + {m_{a4}}

The application deployment time *w_A_k__* from the Eq. (12) accounts for the time spent by the application before all its services are placed on the chosen resources. In the case when one of the services *a*_*i*_∈ *A*_*k*_ cannot be placed in the current fog colony, the whole application needs to be delegated to the neighbor fog colony. Therefore, additional expected deployment time \overline T _{{w_N}}^t can appear. To define whether this additional deployment time affects *w_A_k__* or not, we introduce the auxiliary variable *y_A_k__*. Let *y_A_k__* = 0 if all services can be successfully placed in the current fog colony, and *y_A_k__* = 1 if at least one service *a*_*i*_ ∈ *A*_*k*_ cannot be placed in the current fog colony, and the whole application needs to be delegated. Therefore, the application deployment time *w_A_k__* is defined as follows:

(19) }{}{w_{{A_k}}} = w_{{A_k}}^t + \overline T _{{w_N}}^t{y_{{A_k}}}

It is clear that \overline T _{{w_N}}^t affects the application deployment time only when *y_A_*k*__* = 1. The closest neighbor fog node has own service placement which can either place all services *a*_*i*_ ∈ *A*_*k*_ in its fog colony or further postpone the execution of the application *A*_*k*_ by delegating it further in the fog landscape. To avoid a ping-pong with an application between the two closest fog colonies, additional criteria can be considered, for example, the rate of successful service execution, free capacities, or a percentage of load on a fog colony. In the current work, if the application cannot be deployed in a fog colony or in a neighbor fog colony before a stated deadline, it is either deployed in the cloud if the service types allow that, or not deployed at all. To calculate the expected deployment time in the closest neighbor colony, \overline T _{{w_N}}^t requires to view forward in time. Therefore, we estimate \overline T _{{w_N}}^t relying on historical data. \overline T _{{w_N}}^t is obtained as the moving average on the latest sampled deployment time }{}T_{{w_N}}^{t - \tau } per service delegated to the closest neighbor fog colony as defined in (20), where 1 − *α*, *α* ∈ [0,1] denotes the discount factor of the moving average, }{}T_{{w_N}}^{t - \tau } is the already passed deployment time of the service delegated to the neighbor fog node *N* during the time *t* − *τ* , and \overline T _{{w_N}}^{t - \tau } stands for the average deployment time in *N* as estimated in *t* − *τ*.

(20) \overline T _{{w_N}}^t = \alpha T_{{w_N}}^{t - \tau } + (1 - \alpha )\overline T _{{w_N}}^{t - \tau }

Next, the container capacity meaning the number of deployed containers, should not be more than *K_f_j__* containers for each of the fog cells and *K*_*F*_ for fog nodes as defined in (21) and (22), because that may cause overload.

(21) \sum\limits_{{A_k}}^A \sum\limits_{{a_i}}^{{A_k}} x_{{a_i}}^{{f_j}} \le \gamma {K_{{f_j}}},\forall {f_j} \in Re{s_{{a_i}}}(F)

(22) \sum\limits_{{A_k}}^A \sum\limits_{{a_i}}^{{A_k}} x_{{a_i}}^F \le \gamma {K_F}

We have to provide the condition that in the case when any of the services in an application cannot be placed in the current fog colony, the application request has to be sent to the neighbor colony as has been described in “Application Management”. For that, we first calculate the number of services placed in the current fog colony and in the cloud:

(23) }{}{n_{{A_k}}} = \sum\limits_{{a_i}}^{{A_k}} \left( {\sum\limits_{{f_j}}^{Re{s_{{a_i}}}(F)} x_{{a_i}}^{{f_j}} + x_{{a_i}}^F + x_{{a_i}}^R} \right),\forall {A_k} \in A

Next, if the total number of services placed in the current colony and in the cloud is less than the total number of services in the application, that means if *n*_*A*_*k*__<|*A*_*k*_|, then the application request has to be sent to the closest neighbor colony, namely, *y_A_k__* = 1, else *y_A_k__* = 0. This conditional constraint is formulated using big-M coefficients (Hooker & Osorio, 1999), and is represented in (24) and (25), respectively:

(24) }{}{n_{{A_k}}} - |{A_k}| \le M(1 - {y_{{A_k}}}) - 1,\forall {A_k} \in A

(25) }{}|{A_k}| - {n_{{A_k}}} \le M{y_{{A_k}}} - 1,\forall {A_k} \in A

Finally, we define that each service *a*_*i*_ can be placed in exactly one computational resource *f*_*j*_, *F*, *N*, or in the cloud *R*, or the whole application request has to be sent to the closest neighbor fog colony:

(26) }{}{n_{{A_k}}} + {y_{{A_k}}} = 1,\forall {A_k} \in A

*Example*. To provide an estimation of the number of variables and constraints in the service placement problem, we consider the following example: An application *A*_1_ with 10 services *a*_1_…*a*_10_ is submitted for execution to a fog colony with a head fog node *F* and two fog cells *f*_1_ and *f*_2_. In this example, the assumption is that the both fog cells *f*_1_ and *f*_2_ and the head fog node *F* are able to execute all service types to which the services belong. For this setting, a service placement problem consists of making a decision for in total 41 decision variables, which are:

- Ten decision variables *x*^*f*_1_^_*a*_1__…__*a*_10___ corresponding to the placement decision of each of the services *a*_1_…*a*_10_ on the fog cell *f*_1_ and accordingly another ten decision variables *x*^*f*_2_^_*a*_1__…__*a*_10___ of each service *a*_1_…*a*_10_ on the fog cell *f*_2_.
- Ten decision variables *x*^*F*^_*a*_1__…__*a*_10___ corresponding to the placement of each service *a*_1_…*a*_10_ placed on the head fog node *F*.
- Ten decision variables *x*^*R*^_*a*_1__…__*a*_10___ corresponding to a propagation decision of each service *a*_1_…*a*_10_ to the cloud resources *R*.
- One decision variable *y*_A_1__ denoting whether the whole application needs to be delegated to the neighbor fog colony.

With regard to the constraints, the first set of constraints deal with CPU, RAM and storage capacities of each device according to (8)–(10), meaning three constraints for each computation device *f*_1_, *f*_2_, and *F*. Secondly, the response time constraint is only one according to the calculations (11)–(20) corresponding to one application *A*_1_. Next, there are three container capacity constraints according to (21)–(22) per each *f*_1_, *f*_2_, and *F*. Afterwards, to calculate whether the application *A*_1_ can be placed in the current fog colony or needs to be delegated to the neighbor fog colony, two additional constraints per application, that is in our case only one application, need to be calculated according to (23)–(25). And the last set of constraints ensures that each service can be placed only on one resource, that means one constraint according to (26). To summarize, the number of variables and constraints depends on the number of resources in the fog landscape, the number of applications requested for execution, and the number of services to be deployed in each application.

### Greedy algorithm

As mentioned above, we provide two service placement algorithms as examples in FogFrame. The greedy algorithm implemented in the framework (see Algorithm 1) is based on finding a first fit device for each service request (Xu, Tian & Buyya, 2017). The idea is to walk over sorted fog devices according to their service types, available resource capacities, and incoming service requests, and check whether a fog device is able to host and deploy a service according to the device’s utilization. If any service in the application cannot be deployed, the whole application is delegated to the neighbor colony. The main benefit of greedy algorithms is that they produce fast and feasible solution (Xu, Tian & Buyya, 2017).

**Algorithm 1:** Greedy Algorithm.

**Input:** Set<Fogdevice> *fogDevices*, Set<TaskRequest> *requests*
**Output:** List<TaskAssignment> *assignments*, List<TaskRequest> *openRequests*
1 *assignments* ← []; *round* = 0;
2 *sortedRequests* ← sortByServiceType(requests)
3 *sortedFogDevices* ← sortByServiceType(fogDevices)
4 **for** *fogDevice* ∈ *sortedFogDevices* **do**
5 **for** *serviceType* ∈ *fogDevice.serviceTypes* **do**
6 **for** *request* ∈ *sortedRequests* **do**
7 **if** *serviceType* == *request.serviceType* **then**
8 *utilization* ← getUtilization(*fogDevice*);
9 *containers *← getContainerCount(*f ogDevice*);
10 **if** *checkRules*(*utilization*) & *containers* < *MAX CONTAINERS* **then**
11 *container* ← sendDeploymentRequest(*fogDevice*, *request*);
12 assignments.add(*fogDevice*, *request*,*container*);
13 sortedRequests.remove(*request*);
14 **end**
15 **end**
16 **end**
17 **end**
18 **if** !*sortedFogDevices.hasNext*() & *round* < *ROUNDS* & *sortedRequests.size*() > 0 **then**
19 *round* = *round*+1;
20 sortedFogDevices.reStart();
21 **end**
22 **end**
23 *openRequests* ← sortedRequests;
24 **return** *assignments*, *openRequests*

The algorithm takes the set of fog devices in the fog colony and the incoming service requests as inputs. Line 1 of Algorithm 1 initializes an empty assignments list and a new counter parameter. This counter enables several tries to place a service in the algorithm. This counter is necessary to account for released resources in the case when the execution of already deployed services has been finished at the time of service placement. Lines 2 and 3 sort according sets by service type in order to assign sensor-related services to fog cells with the highest priority because sensor equipment is available only there, for example, a service sensing temperature can be placed only on the device with a temperature sensor attached. If any service can be executed both on the fog device and in the cloud, the service is assigned to the fog device if available.

Lines 4 to 6 start the loops over the sorted fog devices and service requests. These loops ensure the placement of equipment-specific services on fog cells first, and if they can be placed, an attempt to place all other services is performed. Otherwise, the whole application has to be delegated to the neighbor fog colony. Line 7 checks if the service type of the service request corresponds to the service types of the fog device. This is necessary because a constraint on matching service types of a service and device is the main constraint in the service placement, otherwise the assignment is not possible. In Lines 8 and 9, the utilization and the number of already deployed containers is requested from the fog device in order to check if there are enough resources in the fog device. This is done in order to check the utilization constraints in Line 10, where the utilization parameters and the number of deployed services are compared to predefined monitoring rules, for example, CPU utilization <80%.

If those parameters are satisfied, a fog device is able to host a service, and a deployment request is sent to the fog device in Line 11. In the event of successful service deployment, the fog device sends the detailed information about the deployed container to the fog node. In Line 12, an assignment consisting of the fog device, service request, and the identifier of the deployed container is created. With this assignment, it becomes possible to keep track of all the deployed services and according containers in a fog colony. A successfully executed service request is removed from the input set in Line 13 making sure the service is deployed exactly once.

Line 18 checks if the outer-most fog device cycle is finished, the provisioning round counter is smaller than the maximal defined number of tries, and if there are still opened service requests. If this is the case, the round counter is increased and the fog device iterator is re-initialized to restart the provisioning with the remaining service requests (Lines 19 and 20). After all the requests are handled or the maximum number of provisioning rounds is exceeded, the created assignments and the open requests are returned (Line 23 and 24). If the open requests list is not empty, then the according application requests are sent to the neighbor fog colony and already deployed services from those applications are stopped.

### Genetic algorithm

The choice to use a genetic algorithm for service placement is based on the popularity of genetic algorithms to solve similar allocation and scheduling problems in cloud environments (Arunarani, Manjula & Sugumaran, 2019; Zhang et al., 2019; Reddy et al., 2020), their lightweight nature, which helps to run genetic algorithms on resource-constrained devices at the edge of the network, as well as our own positive experience with using genetic algorithms to find solutions to service placement problems in fog computing (Skarlat et al., 2017a).

Genetic algorithms allow to browse a large search space and provide a viable qualitative solution in polynomial time (Yoo, 2009; Yu, Buyya & Ramamohanarao, 2009; Ye, Zhou & Bouguettaya, 2011). One iteration of a genetic algorithm applies the genetic operators of selection, crossover, and mutation on a generation of solutions of an optimization problem (Whitley, 1994). In our case, the optimization problem is to make a decision about service placement on fog resources, i.e., to produce a service placement plan. A generation consists of individuals represented by their chromosomes. Each chromosome denotes one solution to the considered problem, in our case a chromosome is one service placement plan. The genetic algorithm starts with the process of selection of individuals for reproduction. For that the fitness function of each chromosome is calculated based on the goal function and constraints of the service placement problem, and the best chromosomes are selected. To create an offspring from the selected individuals, the crossover operator swaps genes of each two chromosomes. In order not to lose the best-performing candidate solutions, some individuals with the highest fitness values of their chromosomes are forwarded to the next generation unaltered, they do not participate in crossover, becoming the elite individuals. After that in order to ensure diversity of the population, the mutation operator changes a random number of genes in some of the chromosomes. As a result, a new generation is evolved consisting from the elite and offspring individuals. The algorithm repeats this process until activation of defined stopping conditions. Different approaches can be applied as stopping conditions, for example, the total number of evolved generations, a tolerance value of the fitness function, or elapsed time. In the following, we describe the concrete implementation of the genetic algorithm in FogFrame.

**Figure 9:**
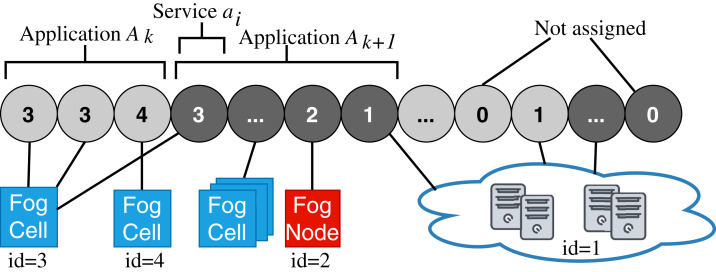
Chromosome representation.

The chromosome representation is a vector corresponding to a service placement plan (see Fig. 9). The length of this vector equals to the total number of services in the requested application. Each gene in a chromosome is an integer number corresponding to the identifier of a fog device or the cloud. When a service cannot be placed at any of the devices in the fog colony or in the cloud, it remains unassigned. In this case, the whole corresponding application will be delegated to the closest neighbor colony. This chromosome representation ensures the placement of all services, meaning that there are no invalid chromosomes. Additionally, the chromosome representation stores necessary data to estimate utilization of devices that includes CPU, RAM, and storage resources of fog devices, and estimated response time of the application.

In the fitness function, we encourage the chromosome if it fulfills the constraints of the system model presented in “System Model” and apply penalties if the constraints are violated (Yeniay, 2005). The constraints of the optimization problem have been divided into three sets which affect the fitness function to different degrees: (i) a set *ψ* of constraints on capacities of CPU, RAM and storage resources of fog devices, (ii) a set *γ* of implicit binary constraints derived from the goal function: conformance to service types, indications if cloud or fog colony resources have to be used, and prioritization of own fog colony resources, and (iii) a set υ causing the death penalty of the chromosome if the service types, container capacities in devices, or deadlines are violated.

Let *c* denote a chromosome. For constraints ∀*β*_*p*_ ∈ *ψ*, if *β*_*p*_(*c*)≤ 0, the constraints are satisfied. If *β*_*p*_(*c*)> 0, then the constraints are not satisfied. These conditions are formalized in (27).

(27) }{}{\delta _{{\beta _p}(c)}} = \left\{ {\matrix{ {0,}  {\quad if{\beta _p}(c) \le 0} \cr {1,}  {\quad if{\beta _p}(c) \gt 0} } } \right.

Similarly, for the Γ set of constraints, if *β*_*γ*_(*c*) = 0, then the constraints are satisfied. If *β*_*γ*_(*c*) = 1, the constraints are not satisfied. For the ϒ constraints, the penalty distance from the satisfaction of ϒ constraints for *c* is defined in (28), where *β*_*υ*_ denotes a constraint, and *δ*_β_*υ*__(*c*) indicates whether a constraint has been violated in the current chromosome *c*: *δ*_β_*υ*__(*c*) = 1.

(28) }{}D(c) = \sum\limits_{{\beta _\upsilon } \in \Upsilon } {\delta _{{\beta _\upsilon }(c)}}

The fitness function is calculated according to (29), where *ω*_β_*p*(*c*)__ is the weight factor of *β*_*p*_ ∈ *ψ*, *ω*_β_*p*(*c*)__ is the weight factor of *β*_*γ*_ ∈ Γ, and *ω*_*p*_ is the penalty weight factor for constraints in *ϒ*. If constraints *β*_*p*_ or *β*_*γ*_ are satisfied in *c*, then *δ*_β_*p*(*c*)__ and *δ*_β_*γ*(*c*)__ become 0, and the according values within the first and the second terms of (29) are added to the fitness function. When the constraints are not satisfied, *δ*_β_*p*(*c*)__ and *δ*_β_*γ*(*c*)__ become 1, and the according values resulting from the first and second terms are subtracted from the fitness function. The third term in the fitness function ensures death penalty *ω*_*p*_*D*(*c*) for having *D*(*c*) other than 0, where the penalty factor *ω*_*p*_ has to be big enough to forbid participation of the worst chromosomes to perform crossover and to create the next generation of individuals. The weight values allow to change the impact of constraints on the fitness function. In this work, weights equal to 1, and the death penalty weight equals to 100,000. When the genetic algorithm is running, the fitness value of chromosomes increases. This happens because less penalties are applied to the chromosomes (Yeniay, 2005).

(29) }{}F(c) = \sum\limits_{{\beta _p} \in \Psi } {\omega _{{\beta _p}}}(1 - 2{\delta _{{\beta _p}(c)}}) + \sum\limits_{{\beta _\gamma } \in \Gamma } {\omega _{{\beta _\gamma }}}(1 - 2{\delta _{{\beta _\gamma }(c)}}) - {\omega _p}D(c)

The genetic operators were determined based on pre-experiments presented in (Skarlat et al., 2017a): We use a 80%-uniform crossover because the genes are integer values, a crossover mixing ratio of 0.5, tournament selection with the arity 2, random gene mutation with a 2% mutation rate, a 20% elitism rate, and a population size of 1,000 individuals.

Regarding the stopping condition, different options exist. Obviously, the fitness value of the fittest individual in the generation has to be a positive number, since a positive fitness value means that there are no death penalties applied to the individual. Time-based stopping conditions of a number of iterations or execution time of the algorithm are not clear to define (Bhandari, Murthy & Pal, 2012). A stopping condition based on improving the variance of fitness function over generations is identified as assuring the algorithm’s convergence.

We use a tolerance value of the fitness function as the stopping condition of the algorithm. It is calculated by dividing the incremental variance of the fitness function values by the maximum fitness value over generations (Bhandari, Murthy & Pal, 2012). The tolerance value of the fitness function is set to *ε* = 0.01, which is enough to obtain the solution and not to converge in local maxima.

With either of the two service placement algorithms presented in “Greedy Algorithm” and “Genetic Algorithm” in place, we are now able to compute a service placement plan in fog nodes in FogFrame.

## Evaluation

In this section, we perform a testbed-based evaluation of FogFrame aiming to show (i) how deployment times of services differ at the edge and in the cloud, (ii) how the services are distributed in the fog landscape by the service placement algorithms according to different service request arrival patterns, and (iii) how much time is spent on producing a service placement plan.

### Implementation and experimental setup

**Figure 10:**
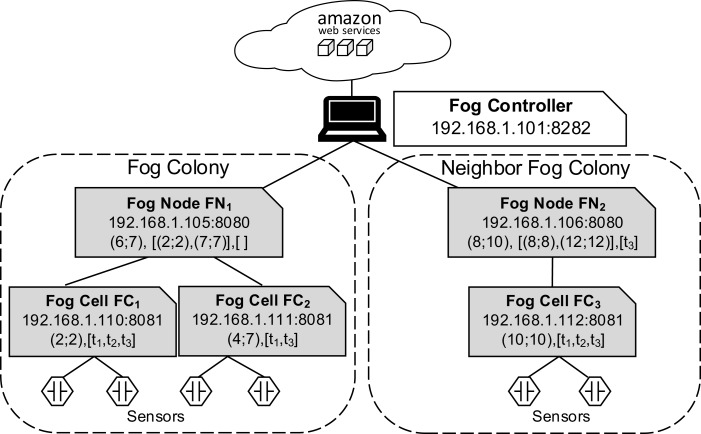
Experimental setup.

**Figure 11:**
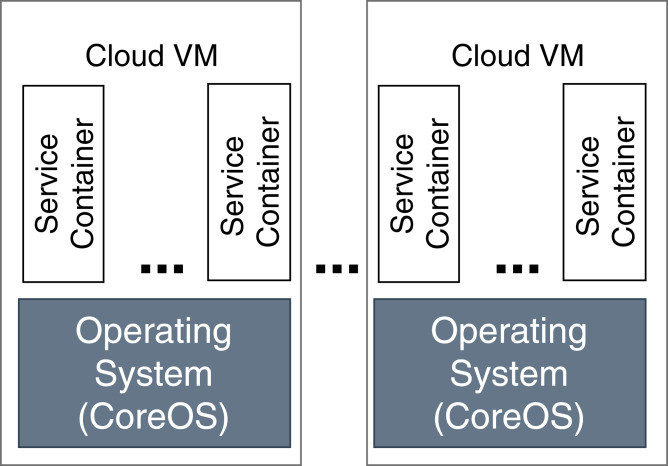
Deployment in the cloud.

In our evaluation scenario, Raspberry Pi computers are used as fog devices (see Fig. 10). Raspberry Pis are based on an ARM processor architecture, which is also used in mobile phones, smart phones, and digital television, to name just some examples. In general, nearly 60% of all mobile devices use ARM chips (Yi et al., 2016). Therefore, Raspberry Pis can be considered as representative when building a fog landscape (Isakovic et al., 2019). Fog nodes and fog cells are deployed on Raspberry Pi 3b+ units (Quadcore 64-bit ARM, 1 GB of RAM), which run with the Hypriot operating system. The detailed setup configuration of FogFrame is described in (Bachmann, 2017). The fog controller is deployed with a Docker container in an Ubuntu 18.04 LTS virtual machine with a 2-Core CPU, 4 GB RAM, which is running on a notebook with Intel Core i7-5600U CPU 2.6 GHz, and 8 GB RAM. The design of the fog controller allows to deploy it in the same manner as fog nodes and fog cells on Raspberry Pis. For public cloud resources, we use Amazon AWS EC2 services, specifically t2.micro VMs with the CoreOS operating system which has a Docker environment setup by default (see Fig. 11).

The FogFrame framework is implemented by means of Java 8 in combination with the Spring Boot framework which provides a convenient persistence handling with Spring Data and Java Persistence API. The framework is available as open source software at Github (https://github.com/softls/FogFrame-2.0). Fog cells and fog nodes are by themselves services running inside their own Docker containers in a Docker runtime environment which is provided by the host operating system of Raspberry Pi units or cloud VMs. Therefore, during service deployment, a problem appears when trying to instantiate other Docker containers in the Docker runtime from inside the Docker containers of the running fog cells and fog nodes. To make it possible for fog cells and fog nodes to start and to stop further Docker containers on the host device, a Docker hook is implemented. The Docker hook provides a communication mechanism from inside the fog cell’s and fog node’s Docker containers into the Docker environment of the operating system of the Raspberry Pi.

Fog nodes *FN*_1_ and *FN*_2_ are connected to the fog controller. The fog colony controlled and orchestrated by *FN*_1_ consists of two fog cells *FC*_1_ and *FC*_2_, which are within the coverage area of *FN*_1_. The fog colony controlled and orchestrated by *FN*_2_ has one connected fog cell *FC*_3_. Temperature and humidity sensors are installed on the Raspberry Pis of corresponding fog cells by the means of GrovePi sensor boards (https://www.dexterindustries.com/grovepi/).

Services of FogFrame intercommunicate via REST APIs. The communication within the testbed is done via a WLAN private network provided by a Linksys Smart WiFi 2.4GHz access point. This access point also acts as a gateway to connect every Raspberry Pi to the Internet. Every component needs to be connected to the Internet since the fog services require the ability to download Docker image data in order to create and deploy services. The private network in which our fog landscape operates is deemed to be secure, and all components of the fog landscape communicate via dedicated API endpoints on certain ports and IP addresses specified in the framework. It is a matter of future work to research other appropriate security mechanisms for fog computing (Tange et al., 2020).

Regarding the virtualization technology, cloud resources are virtualized by the means of VMs. As discussed in “Introduction”, VMs are not a good choice for fog devices, so for them, we use Docker containers instead. The implemented service deployment and execution mechanisms for the cloud resources and fog colonies are different, since the hardware used in these environments differs. In order to use Docker containers in fog colonies, the base images of containers have to be compatible with the ARM processor architecture of Raspberry Pis, and accordingly in order to use Docker containers in cloud resources, the base images of those containers have to be compatible with the processor architecture of the cloud-based VMs.

**Figure 12:**
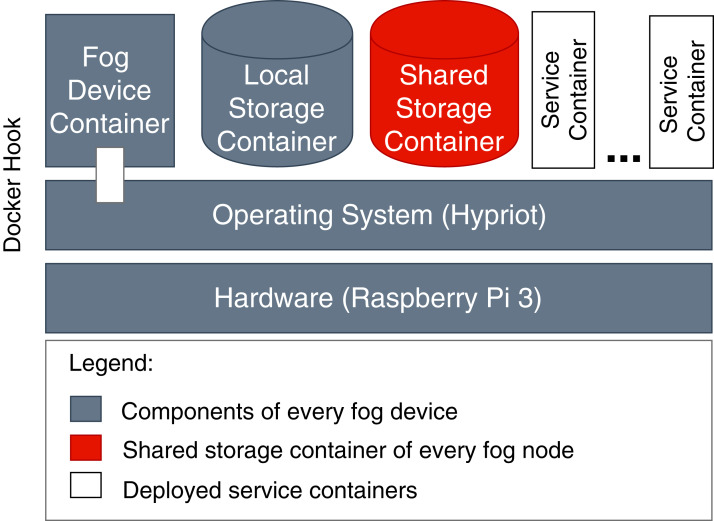
Components of fog devices.

To store the images of the single services, we apply different independent storage solutions. Service images of services to be executed in the cloud are stored in an online repository Docker Hub (https://hub.docker.com/), which is accessible by cloud VMs. For service images of services to be executed in fog colonies, we implement a shared storage that contains the shared service registry (see “System Architecture”). Such distribution is necessary because in order to be executed on specific fog devices or in the cloud, each service image has to be compiled according to the processor architecture of that computational resource. In the case when services need to be executed in the cloud, they are downloaded via a link provided with a service request. This is necessary because we do not consider having a pre-configured pool of idle cloud resources with already stored service images. In the case when services need to be executed on fog devices, service images are sent to a fog node together with the initial application request. In fog colonies, applications (or more precisely: their services) are distributed between different fog devices, therefore every device needs access to the service images. This is ensured by the *shared storage* (see Fig. 12). It has to be noted that there is no limitation on where to host a shared storage because direct IP communication is established in the framework.

In our experimental setup, each application consists of a number of services of certain service types, and is characterized by its makespan duration and a deadline on the deployment and execution time, as has been formally described in “Service Placement”. For that, we have defined and implemented three possible service types: Services of type *t*_1_ get data from temperature and humidity sensors and are executable only on fog cells because services of this type need sensor equipment; services of type *t*_2_ and *t*_3_ simulate processor load and are executable either on fog devices or in the cloud. We have also developed a dedicated service to be deployed and executed in the cloud which receives sensor readings and writes them to a cloud database.

### Metrics

To assess how much time is spent on deployment in the cloud and at the edge of the network, we calculate the service deployment time. This metric is separately evaluated for the cloud and for fog colonies. The service deployment time in the cloud depends on whether there have already been free VMs running, or if a new VM has to be started. Furthermore, the deployment time depends on the availability of the required service image. In case no free VM is available, the service deployment time in the cloud equals the sum of the VM booting time, the time to pull the service image, and the startup time of the Docker container in that VM. If a free VM is available but the service image has to be pulled, the service deployment time in the cloud equals the sum of the time to pull the service image and the startup time of the Docker container, meaning a cold start of the container. When both a free VM and the required service image are available, the service deployment time in the cloud equals the startup time of the Docker container.

The service deployment times in fog colonies differ from the ones in the cloud because in the fog colonies no VMs have to be started before deploying the service containers. If a service image is not available locally on the according fog device, the deployment time equals the sum of the time to pull the service image and the startup time of the Docker container. If a service image is available, the service deployment time equals the startup time of the Docker container.

In order to show how services are distributed in the fog landscape by different service placement algorithms, we record the number of deployed services (containers) in each device in fog colonies and in the cloud. We also record the total deployment time of each scenario. Furthermore, we record the computational time of producing a service placement plan depending on the number of service requests.

In order to show how services are recovered in the case of a failure or migrated in the case of device overload, we record average metrics of recovery time per service and time to migrate a service due to a device overload. We also record how fast the framework reacts to a new device appearing in the fog landscape and deploys services on it.

In the area of cloud computing and accordingly in fog computing, experiments are prone to variations due to a multitude of factors, for example, hardware differences and network quality. Some of the factors cannot be mitigated, instead, a sufficient number of repetitions of experiments ensures that their results are not received due to a chance, but have a sound statistical confidence (Papadopoulos et al., 2019). In order to record the mentioned metrics and show the distribution of results, we execute each experiment ten times. Through ten repetitions per experiment it was noted that the results did not show large variations, and it is a reasonable figure for the number of repetitions.

### Experiments

#### Assessment of deployment time

In this experiment, we show how deployment times of services differ in the cloud and fog colonies. The application used for this scenario is a cloud-edge data processing application with an equal number of 15 service requests to be deployed in the cloud and on the fog devices. The makespan duration of the application is 1 min. The experiment is repeated ten times.

#### Service placement with different arrival patterns

**Figure 13:**
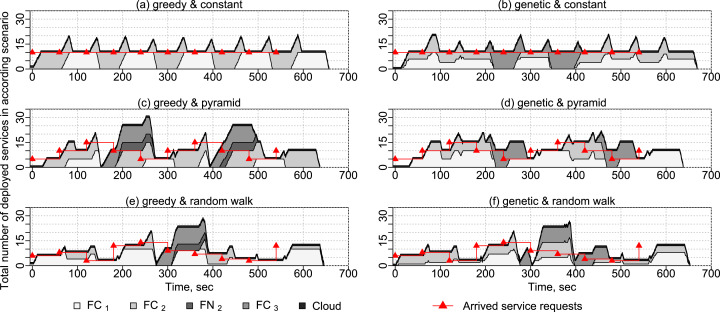
Results of experiments with different placement algorithms and arrival patterns.

In this experiment, we show how services are placed on different fog devices in time. For this experiment, we use applications with different numbers of service requests according to different service request arrival patterns: constant, pyramid and random walk (see Fig. 13). Each application has a makespan duration of 1 min and a deadline on deployment and execution times of 3 min. The arrival patterns are shown along with the representative results of experiments. We evaluate the two service placement algorithms presented in “Service Placement”—the greedy algorithm and the genetic algorithm. One VM and one Docker container with the service to write sensor data into the cloud database are started before the experiment to receive sensor data. The experiment is repeated 10 times.

#### Computational time

In this experiment, we submit applications with different numbers of service requests to fog node *FN*_1_, and observe how much time is needed for the genetic algorithm to produce a service placement plan in each case. The experiment is repeated ten times for 5, 10, 15, 25, 50, 100, 200 and 400 services, in order to show how the genetic algorithm’s computational time increases if the number of services grows.

Computational times are recorded only for the genetic algorithm because, as has been described in “Greedy Algorithm”, in the greedy algorithm the deployment happens immediately when appropriate edge devices are checked for placement, while in the genetic algorithm a service placement plan is generated, and only afterwards the services are deployed.

#### Migration of services

In this set of experiments, we implement a set of instructions and submit them to the fog colony with the head *FN*_1_. As a first step, an application is submitted to the fog node, undergoes the service placement, and is deployed in the fog colony. Fog cell *FC*_1_ in the fog colony experiences a failure and loses its connection, which is simulated by executing a command of stopping the corresponding Docker container of the fog cell. This event triggers the device failure service to calculate a new service placement of all services that were deployed in the failed fog cell. Some services are redeployed on *FC*_2_ and some are delegated to the cloud. Afterwards, *FC*_1_ is restarted as a new fog cell *FC*′_1_ to simulate device discovery in the fog colony. When the discovery service detects this new fog cell, it immediately triggers a new service placement to migrate all the services that are running in the cloud and some services from the devices in the fog colony loaded to the maximum capacity. The corresponding VM in the cloud becomes free of services and therefore is automatically stopped. In order to simulate overload of a fog device, we open several SSH connections to the fog cell *FC*_2_ each running resource-intensive tasks. The device overload service detects this event and migrates one by one randomly-chosen services from the fog cell to another resource in the fog colony (to fog cell *FC*′_1_) until the overload is eliminated. The experiment is repeated 10 times.

### Results and Discussion

#### Assessment of deployment time

In the application executed in this experiment (see “Assessment of Deployment Time”), there are 30 service requests. Out of these, 15 need to be deployed in the cloud, and 15 need to be deployed in the fog colonies. When services have to be deployed in the cloud, in addition to the high start-up times of VMs, VMs do not have previously stored or cached data, for example, previously used service images. Therefore, for the cloud VMs, Docker images need to be pulled every time. In contrast, fog devices download service images only once and then reuse them whenever needed as the images have been cached. As can be seen in Table 2, there is a significant difference between the measured service deployment times at the edge and in the cloud. The average total deployment time at the edge is at about 29.76 s (*σ* = 4.08), whereas the average total deployment time in the cloud is 209.42 s (*σ* = 20.41). The Docker image pull times of the VMs have been also recorded in this experiment. It takes on average 32 s (*σ* = 0.01) to pull and start the docker container in the cloud (see Table 2).

**Table 2:** Assessment of deployment time (in seconds).

	max	min	*μ*	*σ*
Total (edge)	41.78	27.22	29.76	4.08
Per service (edge)	2.78	1.81	1.98	0.27
Total (cloud)	251.03	180.75	209.42	20.41
Per service (cloud)	16.74	12.05	13.96	1.36
VM startup	72	40	48	12
Image pull (start)	33	32	32	0.01
Total	278.94	210.42	239.18	18.77

To summarize the outcome of this experiment, we compared the deployment times in the cloud and in fog colonies. The deployment time in the cloud is higher than the deployment times in the fog colonies because of additional latency, VM start-up time, and service image download time before instantiating according containers. In fog colonies, the shared service registry ensures caching of all available service images at the time fog devices enter the fog colony. In the already running VMs in the cloud all necessary service images are already cached and can be reused. However, each additional new VM in the cloud requires according instantiation time and service image download time and caching time. Having a pre-configured pool of idle cloud resources with cached service images in the same manner as the shared service registry in fog colonies would negate the whole concept of on-demand resources of the cloud. Therefore, cloud resources can be an on-demand addition to the fog landscape, but is not suited to be the only computational resource for latency-sensitive IoT applications.

#### Service placement with different arrival patterns

If applying the greedy algorithm presented in “Greedy Algorithm” and different arrival patterns (see Fig. 13), services are placed on fog cells to the maximum capacity according to the available utilization parameters. If both fog cells in the first fog colony (see Fig. 10) are loaded to the maximum capacity, and a new application request arrives with some services which need sensor equipment, the deployment in the own fog colony becomes impossible, and therefore such a request is delegated to the closest neighbor fog colony.

In the genetic algorithm discussed in “Genetic Algorithm”, the requested applications are distributed in a more balanced way between fog colonies and the cloud. Fog devices are loaded less than to the maximum capacity. By delegating applications between the colonies and distributing single services on different fog devices, the genetic algorithm placement spreads the load on the resources in fog colonies more efficiently, which may be crucial if additional application requests are submitted and their services need specific equipment, for example, temperature and humidity sensors.

In the pyramid and random walk arrival patterns, both algorithms perform almost alike due to the fact that even if the greedy algorithm loads one fog cell for all services in the application request, in most of the application requests the workload is less than the maximum capacity of the available fog devices. However, as can be seen in Fig. 13, the load on fog devices is nevertheless more distributed if the genetic algorithm is used to compute a service placement plan.

To summarize the results of this experiment, the genetic algorithm performs better with regard to distributing service requests within fog colonies. This makes it possible for newly requested applications to be placed on the necessary resources. While the greedy placement does not involve cloud resources, the genetic algorithm spreads the load between the fog colonies and the cloud. In the closest neighbor fog colony, the deployment time per service is longer as there is only one fog cell connected, the fog node’s resources also execute services, and services are deployed sequentially. One particular positive aspect in the genetic algorithm’s placement plan is that the resources in the fog landscape are not close to overload, which gives more opportunities for newly requested services to be deployed. The results of the experiment are summarized in Table 3.

**Table 3:** Experiment results overview.

Metrics	Algorithm	Constant	Pyramid	Random
Deployment time per scenario (sec)	Greedy	694.10	643.50	663.67
		(*σ* = 70.92)	(*σ* = 9.54)	(*σ* = 19.99)
	Genetic	684.00	644.88	654.90
		(*σ* = 16.34)	(*σ* = 9.00)	(*σ* = 7.06)
Service deployment time (sec)	Greedy	3.67	2.18	2.90
		(*σ* = 1.85)	(*σ* = 0.25)	(*σ* = 1.43)
	Genetic	2.08	2.12	2.00
		(*σ* = 0.32)	(*σ* = 0.27)	(*σ* = 0.21)
Service deployment time in the neighbor fog colony (sec)	Greedy	2.52	2.50	2.98
		(*σ* = 0.60)	(*σ* = 0.24)	(*σ* = 0.30)
	Genetic	2.12	2.23	2.38
		(*σ* = 0.13)	(*σ* = 0.27)	(*σ* = 0.56)
Service deployment time, cloud (sec)	Genetic	3.25	3.97	3.19
		(*σ* = 0.18)	(*σ* = 0.13)	(*σ* = 0.92)
Number of services delegated	Greedy	18 (*σ* = 4)	24 (*σ* = 4)	16 (*σ* = 6)
	Genetic	24 (*σ* = 9)	22 (*σ* = 4)	13 (*σ* = 4)

#### Computational time

**Figure 14:**
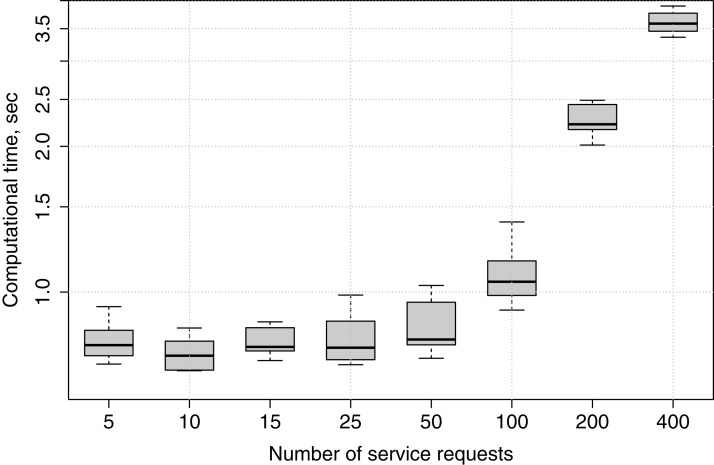
Computational time of producing a service placement plan by the genetic algorithm.

The measurements in this experiment show that the computational time of producing a service placement plan using the genetic algorithm is less than a second on average in all the cases below 50 service requests (see Fig. 14). After that, an increase is observed, however, that increase is still within reasonable boundaries: a service placement plan for 200 and 400 service requests is produced on average in 2.26 s (*σ* = 0.16) and 3.64 s (*σ* = 0.19), respectively.

The relatively small spread of whiskers and box sizes in Fig. 14 show that the computational time is rather stable for the specified number of services. The results mean that increasing considerably the number of services to be deployed affects the computational time, as the genetic algorithm calculates fitness values for each chromosome in each population as well as estimations to response times of applications and fog landscape resource utilization.

#### Migration of services

**Figure 15:**
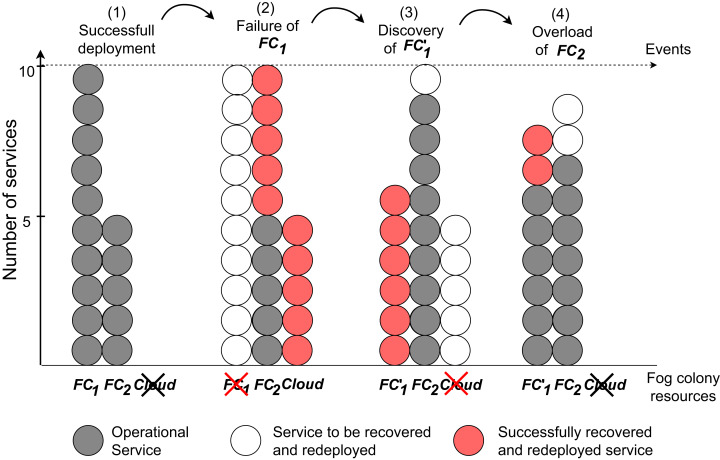
Deployment and migration of services to different resources due to the runtime events.

In this scenario, we consider four distinct events: (1) successful deployment and operation of an experimental application consisting of 15 services: Five services of each type *t*_1_, *t*_2_ and *t*_3_, (2) failure of *FC*_1_ and as a consequence recovery of all the services which were running on *FC*_1_ to *FC*_2_ and cloud, (3) discovery of the new fog cell *FC*′_1_ and migration of services from the cloud and *FC*_2_ to *FC*′_1_, and (4) overload of *FC*_2_ and as a consequence migration of services from *FC*_2_ to other resources one by one until the overload is eliminated. The execution of the scenario is shown in Fig. 15. During the execution, we recorded the average time-to-recover after failure per service, time-to-redeploy during the overload, and time of device discovery of how fast the device is detected in the fog colony and necessary services are migrated onto this device. The insights received from this experiment show that the main setback in the scenarios relying on the cloud is the could itself, namely as a result of request timeouts: connection timeout, request timeout, and read timeout. These parameters of requests to the cloud and the number of retries need to be fine-tuned according to the application needs. When such timeouts occur, the time for leasing and releasing of cloud resources can be affected. These problems have been mitigated in the fog controller’s cloud connector. The results of the execution are: After the failure of *FC*_1_, all 10 services that were deployed there are successfully recovered in the cloud and in *FC*_2_. The average total time-to-recover of 10 services is 23.652 s (*σ* = 1.49). The average of the time-to-recover per service after the failure is 2.35 s (*σ* = 0.14). The average discovery time of a new fog cell from the point of time it is detected by the discovery service until all necessary services are migrated is 18.79 s (*σ* = 2.78). The average time-to-redeploy a service after the device overload is 5.37 s (*σ* = 2.07).

### Opportunities for future work

While the conducted experiments have shown that FogFrame is able to serve its purpose to provide coordinated control for a fog landscape and to execute applications, there are nevertheless some limitations with regard to the evaluation and the framework itself.

Currently, we do not consider delegating a single service from an application to a neighbor fog colony. This is done in order to avoid high intra-application latency and because of the necessary coordination between fog colonies on the level of service requests. It remains a matter of future work to identify approaches to perform service execution tracking in a complete fog landscape.

In the evaluation, we implemented the types of services to be executable in the framework as described in “Implementation and Experimental Setup”. In reality, service types can be different depending on their purpose and necessary equipment. Nevertheless, the applied service types already show how different types can be deployed in the cloud and in the fog by applying FogFrame.

A considerable limitation is that software to be executed on fog devices, meaning that both internal services of FogFrame and applications submitted by the users, have to be adapted or reimplemented according to the processor architecture of the according hardware, for example, according to the ARM processor architecture in a Raspberry Pi. For example, dynamic programming solvers which can be used in service placement, for example, IBM CPLEX solver, JAVA ILP, or Gurobi, do not provide library distributions runnable on the devices with the ARM processor architecture so far. This problem is not specific to our work, but is a common problem when using fog infrastructure.

In this work, we have considered one fog landscape and communication and application execution within and between its fog colonies and the cloud. However, it is a promising research topic to investigate a meta control layer in the fog to allow not only communication between fog colonies, but also between multiple fog landscapes. This would allow optimization of the topologies of the fog landscapes with regard to proximity, efficiency, and volatility of fog resources.

According to state-of-the-art surveys (Kashani, Ahmadzadeh & Mahdipour, 2020; Afzal & Kavitha, 2019), other methods for service placement and migration could be implemented to tackle the volatility of the fog landscape (Nair & Somasundaram, 2019).

Since fog computing is still a quite recent and developing research area, proper security mechanisms remain a challenge. In this particular work, the private network in which the fog landscape operates is deemed to be secure, and all components of the fog landscape communicate via dedicated API endpoints on certain ports and IP addresses specified in the framework. Additionally, ingress and egress rules can be set up on each device allowing only specific framework-related interactions. Other software and hardware security mechanisms for fog computing need to be further investigated (Tange et al., 2020).

Another promising improvement is adopting the recent ETSI standard on context information management and NSGI-LD API metadata (ETSI GS CIM 009, 2019) within the implemented API of the FogFrame framework. This would allow to unambiguously use geographic location queries, temporal data, and linked data coming from different sources.

## Related Work

To the best of our knowledge, already existing contributions in the area of fog computing are often evaluated by the means of simulators along with artificially generated data, since there is still a lack of research testbeds which could be used to evaluate different mechanisms in fog computing. Also, with regard to service placement, many existing approaches simply assume that a fog landscape is already available and can be used. Therefore, in this section, we focus on the works which provide concrete implementations of fog architectures. After that, we devote our attention to different service placement methods.

### Architectures

Battulga, Miorandi & Tedeschi (2020) introduce the *FogGuru* platform for fog computing implemented via a real-world testbed. Their representative fog landscape is built out of five Raspberry Pis united in a cluster cloud tier. The cloud is utilized to host a static service to process sensor data. Their system utilizes a publish-subscribe mechanism to push sensor data through a stream processing system and further into the cloud tier. For orchestration purposes, Docker Swarm is used, and one of the five Raspberry Pi units is used as a Swarm Manager. Unlike our work, the work of Battulga et al. shows how to utilize a publish-subscribe mechanism in fog computing. Their testbed is static, unlike ours, where we explicitly tackle runtime operation events in the fog landscape and migrate necessary services when needed.

In the work of Mahmud, Ramamohanarao & Buyya (2019), alongside with a simulated environment via iFogSim, the authors implement a small static testbed of eight smartphones as IoT devices and five standard computers that act together as a fog cluster of resources interconnected with LAN. This fog cluster operates within their framework called *FogBus*. Service placement is based on a time-optimized QoS-based policy and follows application deadlines. For this, a heuristic evolutionary algorithm to create a placement map of applications onto available resources in the fog cluster is applied. To address possible failures of resources within the fog cluster, in their work a replication mechanism is provided. Compared to their work, our proposed framework ensures cooperation between different fog colonies. The fog landscape automatically detects if devices appear or disappear from the fog landscape, and places, migrates, and optimizes services accordingly.

Another Raspberry Pi-based testbed called *piFogBed* is presented by Xu & Zhang (2019). Their system has a coordinator deployed on a standard computer that contains user management functionality, a device allocator for service placement of user applications, a container manager to save service images to DockerHub, a network simulator and an application execution controller. Fog nodes are deployed on four Raspberry Pi units and execute applications. Service placement is implemented in a set of policies that ensure the utilization of closest fog nodes until their capacities reach a certain threshold and taking into account bandwidth and delay constraints. Their work is a good example of holistic and detailed experiments. Compared to the work of Xu and Zhang, we consider multiple fog colonies that utilize a decentralized service placement for application execution.

An interesting combination of blockchain and fog computing technologies is proposed in the work of Cech, Großmann & Krieger (2019). Their decentralized architecture is based on MultiChain nodes embedded in more powerful fog cells and a P2P network. This P2P network overlay provides a distributed data store to share sensor data between resources in the fog landscape. The authors implemented a testbed with three Raspberry Pi units connected to a standard computer. Docker Swarm was used for orchestration of applications. Blockchain functionality is implemented by a Docker image of blockchain based on the MultiChain framework. In our work, we focus on the fog landscape itself, on how it is formed, how the communication is performed between different fog colonies, and how the volatility is tackled. The promising mechanism of Cech and Krieger could be applied for a distributed data store, as well as to enable tracking of application execution around the fog landscape.

He et al. (2018) propose a simulated fog computing model introducing static dedicated and volatile *opportunistic* fog landscapes as well as fog masters and fog workers as the main entities in fog landscapes resembling our fog nodes and fog cells. Similarly to our approach, the presented model enables multiple fog masters in one fog environment. In the pairing mechanism, He et al. consider invitations from fog workers in order to enter the fog landscape, while in our work fog cells and fog nodes perform self-announcement. Even though their system is simulated, He et al. provide very interesting insights on interactions within different fog environments.

Tsai et al. (2017) implement a distributed analytics platform based on Raspberry Pis using TensorFlow and Kubernetes. Their testbed consists of a centralized server and up to four fog devices connected by an Ethernet switch. The applications are split into small operators by TensorFlow, which is comparable to the way we assume applications to be composed out of services. Kubernetes controls and monitors the fog landscape, checks the available resources, and deploys Docker containers of operators on-demand. In contrast, in our work, we have developed an own distributed management system for the fog landscape which is not centralized like Kubernetes. Fog nodes are lightweight compared to Kubernetes, nevertheless they perform all necessary functionalities in own fog colonies, namely, monitoring, management and orchestration of own fog colonies.

Yigitoglu et al. (2017) introduce the fog computing framework *Foggy*. Comparable to FogFrame, the according testbed is implemented based on four Raspberry Pi units. Compared to our work, Yigitoglu et al. do not consider multiple fog colonies and a hierarchical fog landscape. Resource provisioning in their work is performed by an orchestration server, which runs on every node in the network, and implements a first fit provisioning method. In our work, we consider forming decentralized fog colonies and communication between them, and adjust service placement to account for the volatile nature of the fog landscape, for example, device discovery, failures and overloads.

We provide a summary of the findings from the related fog computing architectures in Table 4. This table provides the insights for each considered work of whether it is implemented or simulated, makes use of VMs and containers, is a centralized solution or distributed, accounts for the communication within the dedicated fog computing environment, and considers the communication between multiple fog landscapes.

**Table 4:** Overview of implementations of fog architectures.

Work	Implemented	VMs	Containers	Centralized reasoning	Distributed reasoning	Intra-fog communication	Inter-fog communication
Battulga, Miorandi & Tedeschi (2020)	**✓**	**✓**	**✓**	**✓**		**✓**	
Mahmud, Ramamohanarao & Buyya (2019)	**✓**	**✓**		**✓**		**✓**	
Xu & Zhang (2019)	**✓**	**✓**	**✓**	**✓**		**✓**	
Cech, Großmann & Krieger (2019)	**✓**		**✓**	**✓**		**✓**	
He et al. (2018)					**✓**	**✓**	
Tsai et al. (2017)	**✓**	**✓**		**✓**		**✓**	
Yigitoglu et al. (2017)	**✓**		**✓**	**✓**		**✓**	**✓**
FogFrame	**✓**	**✓**	**✓**		**✓**	**✓**	**✓**

### Service placement

So far, we have discussed contributions to the management and communication in fog landscapes. In the following, we consider related work in the area of resource provisioning and service placement in the fog. Notably, literally hundreds of studies on service placement in the fog have been presented in recent years (Bellendorf & Mann, 2020; Salaht, Desprez & Lebre, 2020; Hong & Varghese, 2019). An interesting systematic review has recently been presented by Kashani, Ahmadzadeh & Mahdipour (2020). It provides comprehensive descriptions, advantages and disadvantages of approximate, exact, fundamental, and hybrid methods of load balancing in fog computing. Most of the methods mentioned in the survey are simulated, and it is promising to implement the mentioned methods in a real-world fog computing environment. Also, some of the cloud computing resource provisioning methods can be adapted onto fog computing. In another recent survey by Afzal & Kavitha (2019), advantages and limitations of existing load balancing methods in cloud computing are considered. These methods can be implemented within the fog controller’s reasoning mechanisms to manage additional cloud resources for fog colonies. Because of the large number of existing approaches, we will focus on the content-wise closest work in the next paragraphs.

Brogi & Forti (2017) introduce the *FogTorch* tool which aims to perform resource provisioning in fog landscapes. FogTorch accepts a fog landscape infrastructure and application specifications as inputs, and calculates a deployment model. The basis for the deployment is QoS-aware service placement. The placement approach preprocesses all input requests to search a map of available resources for each service in each request, backtracks the results of preprocessing to guarantee the deployment of all services, and applies a heuristic fail-first algorithm to ensure the deployment of those services having fewer compatible nodes and more demands in resources. In contrast, in our work, we implement FogFrame along with our own resource provisioning mechanisms, and apply it in a real-world Raspberry Pi testbed. The integration of FogFrame with FogTorch may become a good opportunity in the future to deal with the reoptimization of network topologies of fog landscapes.

Xiao & Krunz (2017) consider an offloading problem in fog computing. Optimization is performed based on power consumption and Quality of Experience (QoE) parameters. Their approach is called *offload forwarding*. In contrast to our work, the authors use different criteria for optimization, namely, QoE and power consumption. This is an interesting approach, and our model may be extended to take into account power efficiency of the fog landscape and QoE inputs from users.

Ni et al. (2017) propose a resource allocation technique for fog computing which is based on Priced Timed Petri nets. In the application model used in their work, a fog application is orchestrated from single services. The time and price for execution of these services differ for each device. The resources for allocation are chosen by the users depending on the information received from the Petri net and their own demands. In our approach, users are not involved in the orchestration component, and the reasoning service reacts to application requests automatically.

Saurez et al. (2016) propose to allocate resources and migrate services in the fog based on two possible triggers: (i) meeting latency constraints and (ii) resolving resource pressure. In contrast, in our work, apart from latency and resource constraints, we take into account QoS parameters, namely, deadlines on deployment and execution of applications.

Nardelli et al. (2019) introduce several heuristic approaches to efficiently identify service placement considering the volatility of computing resources. They simulate different network topologies and sizes of fog infrastructure. In their work, several meta-heuristic algorithms are implemented: a greedy first fit, tabu search and local search algorithms. According to findings of Nardelli et al., the greedy first fit algorithm is the fastest, however with the worst quality, whereas the local search heuristics shows the best performance trade-off. In our work, we apply two heuristic algorithms – a greedy and a genetic algorithm, for service placement in a real-world fog landscape. We also introduce different placement mechanisms to account for device discovery, failures and overloads.

Mseddi et al. (2019) introduce service placement implemented by the means of particle-swarm optimization, a greedy algorithm, and an exact optimization. The goal of their placement is to maximize the number of executed applications adhering to their time constraints. According to their results, Mseddi et al. state that the particle-swarm optimization yields high resolution times and is not viable in fog computing environments. Their greedy algorithm aims to minimize the distance and delay between used fog resources taking into account their utilization. In contrast, our greedy algorithm aims to maximally utilize a fog colony adhering to QoS and capacities of available resources as well as to the types of services. In general, the service placement approach in our work differs from the work of Mseddi et al.: it considers multiple fog colonies and offloading of applications as well as contains separate policies to tackle operational events in a fog landscape.

In the work of Abedi & Pourkiani (2020), the authors introduce a service placement algorithm based on an artificial neural network aimed to minimize response times of applications while distributing them in the fog landscape. Their approach is simulated with MATLAB, and therefore it is not clear how long it takes to produce such a neural network in the real world. It has to be noted, that this approach, as well as any other machine learning (ML) model, requires considerable volume of training data. This means that before any neural network can be created, other placement algorithms or service placement policies have to be used to historically record those placement decisions to receive a viable training dataset.

Mostafa, Ridhawi & Aloqaily (2018) also implement an artificial neural network in a simulated environment to make placement predictions based on historical placement data. The algorithms in our work could provide a basis for training data and eventually be substituted by ML models. An interesting recent survey (Abdulkareem et al., 2019) discusses these problems and in general areas where ML can be applied in fog computing: ML for specific IoT service implementations and ML for decision making in resource provisioning.

In general, the considered contributions differ from our work in terms of the system model for service placement in the fog landscape, parameters which are included in this model, and algorithms to provide a solution for this model.

In our previous work (Skarlat et al., 2016; Skarlat et al., 2017a, Skarlat et al., 2017b), we model a conceptual fog landscape and an IoT application to be executed by the means of fog resources. Based on these preliminaries, we formulate the fog service placement problem, which aims to maximize the utilization of fog resources and the adherence to QoS parameters. We simulate a fog landscape by the means of *CloudSim* and *iFogSim* (Gupta et al., 2017) and solve the fog service placement problem by a first fit algorithm, a genetic algorithm, and an exact optimization method. In more recent work (Skarlat et al., 2018), a high-level overview of the architecture of FogFrame is presented.

In contrast, in this paper, we provide the design details and the workflows in a fog landscape, eliminate the usage of simulators and implement a representative real-world Raspberry Pi-based testbed with the FogFrame framework. The framework (i) introduces mechanisms to create a fog landscape and account for its volatile nature, (ii) provides decentralized application execution in multiple fog colonies, (iii) discusses communication mechanisms between different fog colonies, (iv) introduces a service placement problem formulation to account for practical issues dealing with delegating and deployment of applications, (v) implements a greedy algorithm and a genetic algorithm to solve the service placement problem, and (vi) reacts to runtime events in the fog landscape and migrates necessary services to balance workload between resources. We extensively evaluate the framework with regard to deployment times of services and utilization of resources.

## Conclusion

In this work, we have designed and developed the fog computing framework FogFrame. The foundation for the framework are lightweight container technologies and loosely-coupled components to provide a stable and fault-tolerant distributed system. In the course of the implementation, we identified and resolved technical issues of how to create a real-world fog landscape based on Raspberry Pi units, which are considered as representative devices for fog computing. We investigated how to instantiate containers in different computing environments and how to store images of services and share those images within the available infrastructure. A considerable part of this work has been devoted to the problem of how to effectively distribute services in a fog landscape. Therefore, we formalized a system model, and implemented a genetic algorithm as well as a greedy algorithm for service placement.

Experiments were conducted to assess deployment times of applications, behavior of service placement algorithms with different arrival patterns of service requests, and computational time depending on the workload to be processed. The genetic algorithm placement distributes services in a balanced way, while the greedy algorithm loads each of the fog devices with the maximum available resource capacity. If there are no sufficient resources in the current fog colony, applications are delegated to the closest neighbor fog colony. The computational time of the genetic algorithm is stable for the specified number of services, however with the considerably increasing number of services to be deployed, the genetic algorithm performs accordingly more computational operations to evaluate each possible solution. Services are successfully recovered and redeployed when fog cells fail or experience overload. Device discovery ensures efficient balancing and horizontal scalability in the fog landscape as well as allows to release cloud resources and other fog cells, which may be intensively used in the fog colony, by migrating necessary services onto discovered devices.

In this paper, we instantiate and work with one arbitrary fog landscape. However, it is a promising research topic to investigate a meta control layer to allow not only communication between fog colonies, but also between multiple fog landscapes. In future work, various components of FogFrame can be substituted or extended, for example, the resource provisioning and service placement methods, or pairing methods. FogFrame has intentionally been designed and implemented in a loosely-coupled manner in order to allow for this. A particular issue to address in the future work is how to scan and reconfigure the fog landscape structure based on different runtime events: device discovery, overloads, and failures. When devices appear and disappear in the fog landscape, the resulting network constellation may become not optimal in terms of latency, bandwidth, network hops, location mapping, and connection preservation. Therefore, optimizing fog landscape topologies is another research challenge. For this, different resource provisioning methods can be developed and integrated into FogFrame. The framework is publicly available (https://github.com/softls/FogFrame-2.0), and is provided to the research community for further extensions and experiments.

## Additional Information and Declarations

### Data Availability

The following information was supplied regarding data availability: The code and configuration files are available at GitHub: https://github.com/softls/FogFrame-2.0.
